# Structures of collagen IV globular domains: insight into associated pathologies, folding and network assembly

**DOI:** 10.1107/S2052252518012459

**Published:** 2018-10-10

**Authors:** Patricia Casino, Roberto Gozalbo-Rovira, Jesús Rodríguez-Díaz, Sreedatta Banerjee, Ariel Boutaud, Vicente Rubio, Billy G. Hudson, Juan Saus, Javier Cervera, Alberto Marina

**Affiliations:** a Department of Biochemistry and Molecular Biology/ERI BIOTECMED, Universitat de València, Dr Moliner 50, Burjassot, 46100 Valencia, Spain; b Instituto de Biomedicina de Valencia, Consejo Superior de Investigaciones Científicas (IBV–CSIC), Jaume Roig 11, 46010 Valencia, Spain; c CIBER de Enfermedades Raras (CIBERER–ISCIII), Spain; d Laboratorio de Reconocimiento Molecular, Centro de Investigación Príncipe Felipe, Eduardo Primo Yúfera 3, 46012 Valencia, Spain; e Departamento de Microbiología, Facultad de Medicina at Universitat de València, Blasco Ibáñez 17, 46010 Valencia, Spain; f Department of Defense, Center for Prostate Disease Research, Bethesda, Maryland, USA; g BioStratum Inc., Durham, North Carolina, USA; h Department of Medicine at Division of Nephrology and Hypertension, Vanderbilt University Medical Center, Nashville, TN 37232, USA; i Center for Matrix Biology, Vanderbilt University Medical Center, Nashville, TN 37232, USA; j Department of Biochemistry, Vanderbilt University Medical Center, Nashville, TN 37232, USA; k Department of Pathology, Microbiology and Immunology, Vanderbilt University Medical Center, Nashville, TN 37232, USA; l Department of Cell and Developmental Biology, Vanderbilt University Medical Center, Nashville, TN 37232, USA; m Vanderbilt Ingram Cancer Center, Vanderbilt University Medical Center, Nashville, TN 37232, USA; n Vanderbilt Institute of Chemical Biology, Vanderbilt University Medical Center, Nashville, TN 37232, USA; o Departamento de Bioquímica y Biología Molecular at Facultad de Medicina y Odontología, Universitat de València, Blasco Ibáñez 15-17, 46010 Valencia, Spain

**Keywords:** collagen type IV, network assembly, (IV)NC1 hexamers, Goodpasture’s disease, Alport’s syndrome

## Abstract

Basement membranes are extracellular structures of epithelia and endothelia that have collagen IV scaffolds of triple α-chain helical protomers that associate end-to-end, forming networks. The molecular mechanisms by which the noncollagenous C-terminal domains of α-chains direct the selection and assembly of the α1α2α1 and α3α4α5 hetero-oligomers found *in vivo* remain obscure. Autoantibodies against the noncollagenous domains of the α3α4α5 hexamer or mutations therein cause Goodpasture’s or Alport’s syndromes, respectively. To gain further insight into oligomer-assembly mechanisms as well as into Goodpasture’s and Alport’s syndromes, crystal structures of non­collagenous domains produced by recombinant methods were determined. The spontaneous formation of canonical homohexamers (dimers of trimers) of these domains of the α1, α3 and α5 chains was shown and the components of the Goodpasture’s disease epitopes were viewed. Crystal structures of the α2 and α4 non­collagenous domains generated by recombinant methods were also determined. These domains spontaneously form homo-oligomers that deviate from the canonical architectures since they have a higher number of subunits (dimers of tetramers and of hexamers, respectively). Six flexible structural motifs largely explain the architectural variations. These findings provide insight into noncollagenous domain folding, while supporting the *in vivo* operation of extrinsic mechanisms for restricting the self-assembly of noncollagenous domains. Intriguingly, Alport’s syndrome missense mutations concentrate within the core that nucleates the folding of the noncollagenous domain, suggesting that this syndrome, when owing to missense changes, is a folding disorder that is potentially amenable to pharmacochaperone therapy.

## Abstract 2

Crystal structures of the noncollagenous domains of the collagen type IV α1–α5 chains provide structural insight into Alport’s syndrome (a genetic kidney disorder) and the first views of the components of Goodpasture’s autoimmune disease epitopes. These structures define key folding motifs for collagen IV scaffolding of basement membranes, support the operation of mechanisms for restricting the self-assembly of noncollagenous domains, and raise the possibility that homohexamers of α1 or α3 chains could occur in nature.

## 1. Introduction   

The extracellular microenvironment plays a pivotal role in tissue genesis, architecture and function. A defining morphological feature of these microenvironments is the basement membrane (BM), an ancient and specialized form of extracellular matrix that is conserved from cnidarians to humans. BMs underlie epithelia and endothelia (Hagios *et al.*, 1998 ▸; Rhodes & Simons, 2007 ▸; Fidler *et al.*, 2017 ▸) and ensheath muscle, fat and Schwann cells (Campbell & Stull, 2003 ▸; Sanes, 2003 ▸; Sillat *et al.*, 2012 ▸; Court *et al.*, 2006 ▸). BMs function as supramolecular scaffolds that compartmentalize and provide structural integrity to tissues, guide cell migration and adhesion, delineate apical–basal polarity and modulate cell differentiation during development (Hynes, 2009 ▸; Pastor-Pareja & Xu, 2011 ▸; Daley & Yamada, 2013 ▸).

Type IV collagen is a major constituent of BMs. This evolutionarily conserved protein forms networks and plays a crucial structural role in the maintenance of BM architecture (Brown *et al.*, 2017 ▸; Fidler *et al.*, 2018 ▸). In addition, it serves as a ligand for cell-surface integrins, thus influencing cell adhesion, migration and differentiation (Wang *et al.*, 2008 ▸). Not surprisingly, mutations in collagen IV cause BM destabilization and tissue dysfunction, and are associated with inborn defects such as Alport’s syndrome and cerebral haemorrhage (Hudson *et al.*, 2003 ▸; Kuo *et al.*, 2012 ▸; Gould *et al.*, 2005 ▸). Furthermore, Goodpasture’s disease results from a selective antibody-mediated aggression to collagen IV, specifically to epitopes localized in the globular noncollagenous domains (NC1s) of collagen IV α-chains 3 and 5 (Hudson *et al.*, 2003 ▸; Cui *et al.*, 2016 ▸). These are two of the six types of collagen IV chains that exist in humans [designated α1–α6(IV)NC1s; in the following the (IV) will be omitted for simplicity]. The Goodpasture epitopes remain to be structurally characterized, but they are considered to be cryptic, becoming immuno-visible as a consequence of solvent exposure or viral infection (Pedchenko *et al.*, 2010 ▸). These collagen IV-related disorders can be deadly as a result of impaired BM function.

Unveiling how collagen IV is organized in BMs seems to be crucial in order to understand the molecular architectures of BMs and to clarify the pathogenesis of BM-related diseases. As previously mentioned, six different types of homologous polypeptide chains of similar lengths (1669–1712 residues) can be found in collagen type IV. These chains (α1–α6), encoded by paralogous genes, are composed of a short N-terminal domain termed the 7S domain (∼25 amino acids), a long central collagenous domain (∼1400 amino acids) and a non­collagenous globular C-terminal domain of around 230 amino acids [abbreviated as (IV)NC1; the (IV) will be omitted in the following; Timpl *et al.*, 1981 ▸; Borza *et al.*, 2001 ▸]. Three α-chains associate into a protomer with a long parallel triple-helical shaft and nonhelical ends. In the collagen IV network found in BMs, different protomers interconnect by end-to-end interactions. At the C-termini, interactions occur between the trimeric NC1 domains of two protomers, forming a hexamer of these domains reinforced by sulfilimine cross-links (Vanacore *et al.*, 2009 ▸). At the N-termini, four protomers interact through their 7S domains, forming a 7S dodecamer reinforced by aldehyde-derived cross-links (Anazco *et al.*, 2016 ▸). In this way, a combination of C-terminal hexameric and N-terminal dodecameric interactions generates the three-dimensional collagen IV scaffold to which other components of the BMs bind (Hudson *et al.*, 2003 ▸; Brown *et al.*, 2017 ▸). Intriguingly, only three distinct protomers, α1α2α1, α3α4α5 and α5α6α5 (abbreviated in the following as α121, α345 and α565, respectively), forming three distinct networks, α121, α345 and α121–α565, are known to occur in BMs (Boutaud *et al.*, 2000 ▸; Borza *et al.*, 2001 ▸; Gunwar *et al.*, 1998 ▸). Previously unrecognized collagen IV α125 chain combinations have recently been found in various cancer cell lines, which form networks of protomers of α1α2 chains and α5-chain homoprotomers (Revert *et al.*, 2018 ▸). The formation of these novel collagen IV networks is dependent on an exportable protein kinase, the Goodpasture antigen-binding protein (Raya *et al.*, 1999 ▸; Revert *et al.*, 2008 ▸, 2018 ▸), pointing to the involvement of the cellular machinery in regulating the organization of collagen IV networks.

The assembly of the collagen IV network involves two distinct stages of α-chain oligomerization. Three α-chains first associate intracellularly, forming protomers, and the protomers then associate end-to-end outside the cell, generating extracellular networks. Hints on the assembly mechanisms were inferred (i) from the crystal structure of the α121NC1 hexamer isolated from natural sources (Sundaramoorthy *et al.*, 2002 ▸; Than *et al.*, 2002 ▸; Vanacore *et al.*, 2004 ▸), (ii) from the ability of recombinant α1NC1 chains to selectively assemble into hexamers (Boutaud *et al.*, 2000 ▸) and (iii) from refolding experiments with NC1 hexamers (Dölz *et al.*, 1988 ▸). Recent studies in one of our laboratories (Cummings *et al.*, 2016 ▸) revealed that the NC1 domains function as recognition modules, directing the selection and assembly of α-chains into protomers and networks. In turn, chloride ions activate a molecular switch present in NC1 domains that triggers the oligomerization of protomers into networks. However, how NC1 recognition modules direct α-chain oligomerization has so far been ignored.

To obtain further insight into collagen IV network assembly and to advance the characterization of the molecular bases of Goodpasture’s and Alport’s syndromes, we generated various NC1 domains *via* recombinant technology and used protein crystallography to identify and structurally characterize the oligomers produced by these domains. Using this approach, while attempting to produce crystals of the α345NC1 heterohexamer that predominates in the BM of the kidney, we solved the three-dimensional structure of the recently reported homohexamer of α5NC1 domains (Revert *et al.*, 2018 ▸). In addition, we crystallized and determined the structures of the homohexamers spontaneously formed by recombinantly produced α1NC1 and α3NC1 domains. Interestingly, when carrying out similar studies with crystals of the α2NC1 and α4NC1 domains, oligomers were spontaneously formed that were architecturally similar to the hexamers observed for the other chains except that they were dimers of tetramers (octamers) or of hexamers, respectively, suggesting the existence of restrictions that limit the oligomers produced *in vivo*. We also validated the use of self-assembled recombinantly produced NC1 domains for structural studies by reproducing *in vitro* the α121 heterohexamer previously obtained from natural sources (Sundaramoorthy *et al.*, 2002 ▸; Than *et al.*, 2002 ▸; Vanacore *et al.*, 2004 ▸). The information gathered provided insights into the structural features involved in the folding, selection and oligomerization of collagen IV chains to form a protomer. Furthermore, we provide the first snapshots of the components of the Goodpasture autoantigen. We also provide insight into Alport’s syndrome, helping to rationalize the structural bases of the effects of the mutations reported in this disease that map to NC1 domains, and concluding that of these mutations, those that are amino-acid substitutions may cause disease by inducing misfolding, thus opening the way to attempting therapy using pharmacochaperones.

## 2. Methods   

### 2.1. Cloning, protein expression and purification   

We obtained a cDNA library from HEK293 cells (a commercial cell line derived from human embryonic kidney) by retrotranscription with SuperScript reverse transcriptase (Invitrogen) of mRNA extracted from these cells using TRIzol (Invitrogen). This cDNA library was used as template for PCR amplification of the α1NC1, α2NC1, α4NC1 and α5NC1 domains using a high-fidelity thermophilic DNA polymerase (*Pfu* polymerase, Stratagene) and the primers listed in Supplementary Table S1. The PCR-amplified coding sequences for these NC1 domains were used in a second round of PCR amplification with additional primers (Supplementary Table S1) to introduce an N-terminal BM40 secretion peptide followed by a FLAG tag preceding the sequences of the indicated NC1 domains (the residues forming each of these domains are indicated in Supplementary Table S2). These BM40-FLAG-tagged domains were then subcloned into pFastBac1 using BamHI and SacI sites for the cloning of α2NC1 and α5NC1, and XhoI and KpnI sites for the cloning of α1NC1 and α4NC1. In the case of α3NC1, the BM40-FLAG-tagged α3NC1 coding sequence was extracted by SacI digestion from the previously reported Fα3ANU-pRC-CMV vector (Gozalbo-Rovira *et al.*, 2013 ▸; Netzer *et al.*, 1999 ▸) and was then subcloned into the pFastBac1 vector.

The resulting pFastBac1 vectors with the indicated inserts were transformed into DH10Bac competent cells and the recombinant baculoviruses were obtained following the instructions provided by the supplier of the baculovirus expression system (Bac-to-Bac Baculovirus Expression System, Invitrogen). The recombinant baculoviruses were used to produce all of the NC1 domains in Sf9 insect cells, as previously described for α2NC1 and α3NC1 (Gozalbo-Rovira *et al.*, 2013 ▸). All purification steps were carried out at 4°C. Supernatants from 1 l cultures underwent ultracentrifugation (1 h, 160 000*g*) and the equivalent of 2 ml column-packed ANTI-FLAG M2 affinity gel (Sigma–Aldrich/Merck) was added. After packing the gel into a column, it was washed with 50 ml 50 m*M* Tris–HCl pH 7.4, 0.15 *M* NaCl. The NC1s were eluted with 10 ml of the same solution supplemented with 0.1 mg ml^−1^ soluble FLAG peptide (DYKDDDDK). The eluted proteins were concentrated and the FLAG peptide was removed by repeated runs of concentration and dilution in elution buffer without peptide using Amicon Ultra-4 10K Centrifugal Filter Devices (Merck–Millipore). Around 1 mg of soluble recombinant protein was usually obtained per litre of culture.

In addition to being produced in the baculovirus/insect-cell expression system, α2NC1 was also obtained in one of our laboratories as an N-terminally FLAG-tagged fusion protein using HEK293 cells for expression and purification, as described elsewhere (Sado *et al.*, 1998 ▸).

### 2.2. Protein crystallization and structure determination   

Crystals of α2NC1 (expressed in HEK293 cells) and of α1NC1, α3NC1, α4NC1, α5NC1 and α121NC1 (expressed in insect cells) were obtained by the sitting-drop vapour-diffusion technique at 21°C using 0.4 µl protein solution (7.4 mg ml^−1^ protein in 50 m*M* Tris–HCl pH 7.4, 0.15 *M* NaCl, except for α3NC1, for which the NaCl concentration was 0.5 *M*) mixed with 0.4 µl of the crystallization solution indicated in Table 1 ▸. For the α121NC1 crystals a 2:1 mixture (in terms of mass) of α1NC1 and α2NC1 chains was used, whereas for the α5NC1 crystals a 1:1:1 mixture of α3NC1, α4NC1 and α5NC1 chains was used. The crystal-harvesting solutions used for cryopreservation are listed in Table 1 ▸. X-ray diffraction was carried out at 100 K using the indicated beamlines and wavelengths (Table 1 ▸) at the European Synchrotron Facility (ESRF), Grenoble, France or the ALBA synchrotron, Cerdanyola, Barcelona, Spain. Crystallographic data sets were processed and scaled using either *MOSFLM* and *SCALA* (*CCP*4 suite; Winn *et al.*, 2011 ▸) or the *XDS* program package (Kabsch, 2010 ▸). Data-collection details and unit-cell parameters are given in Table 1 ▸. Phases were obtained for all of the crystals by molecular replacement with *Phaser* (McCoy *et al.*, 2007 ▸) using the structure of one α1NC1 domain of the bovine α121 crystal structure as a search model (PDB entry 1t60; Vanacore *et al.*, 2004 ▸). For the α3NC1 and α121NC1 crystals, initial data processing with *SCALA* indicated 39–40% twinning in both cases. By alternating cycles of refinement with *REFMAC*5 (Murshudov *et al.*, 2011 ▸) with manual model building using *Coot* (Emsley *et al.*, 2010 ▸) and, in the case of the α3NC1 and α121NC1 crystals by applying twinning refinement as implemented in *REFMAC*5, final models at 1.8–2.8 Å resolution, depending on the crystal, were obtained (see Table 1 ▸). The crystals exhibited good quality-control parameters and excellent stereochemistry (the Ramachandran plot distributions of favoured/allowed/dis­allowed residues for α1NC1, α2NC1, α3NC1, α4NC1, α5NC1 and α121NC1 were 97.7/2.3/0, 97.9/2.1/0, 97.7/2.3/0, 95.3/4.5/0.2, 97.8/2.2/0 and 96.3/3.7/0%, respectively). The structures and structure factors have been deposited in the PDB as entries 5nay, 5nb2, 5nb0, 5nb1, 5naz and 5nax for the α1NC1, α2NC1, α3NC1, α4NC1, α5NC1 and α121NC1 crystals, respectively.

### 2.3. Residue numbering for NC1 domains   

We have followed the convention used for the previously reported structures of α121NC1 heterohexamers obtained from BMs (PDB entries 1li1, 1t60 and 1t61; Than *et al.*, 2002 ▸; Vanacore *et al.*, 2004 ▸). Correspondences with the residue numbering for each complete α-chain are provided in Supplementary Table S2.

### 2.4. Other methods   

Structure-based alignments, structural superimpositions and analysis of protein contacts were performed with *MUSTANG* (Konagurthu *et al.*, 2006 ▸; Papadopoulos & Agarwala, 2007 ▸), *SUPERPOSE* and *NCONT* (*CCP*4 suite, Winn *et al.*, 2011 ▸), respectively. Figures were produced using *PyMOL* (http://www.pymol.org).

Size-exclusion chromatography was performed at 20°C using a Superdex S200 (10/300) column fitted to an ÅKTA 900 FPLC system (column and instrument from GE Healthcare, Barcelona, Spain) using 50 m*M* Tris–HCl pH 7.4, 0.15 *M* NaCl at a flow rate of 0.5 ml min^−1^ as a running buffer. Individual NC1 chains were incubated overnight at 20°C to allow oligomerization before injection, continuously monitoring the absorbance at 280 nm in the effluent.

Surface plasmon resonance (SPR) assays were performed using a Biacore T100 instrument (GE Healthcare). α3NC1 or α5NC1 was immobilized on CM5 sensor chips (GE Healthcare) using amine-coupling chemistry by passing 1 µg ml^−1^ solutions of either NC1 chain in 10 m*M* sodium acetate pH 3 to attain a level of attachment of ∼1000 resonance units (RU). The assays were performed at 25°C by passing a solution consisting of 10 m*M* Tris–HCl pH 7.4, 0.15 *M* NaCl, 3 m*M* ethylene­diaminetetraacetic acid, 0.005% surfactant P20 (GE Healthcare) including the indicated concentrations of α3NC1 or α5NC1 at a flow rate of 10 µl min^−1^ for 300 s, followed by a 300 s dissociation time with protein-free fluid. Between runs, the chip was regenerated by washing with 20 m*M* NaOH at a flow rate of 75 µl min^−1^ for 20 s followed by re-equilibration in running buffer. *BIAevaluation* 2.0.3 (GE Healthcare) was used to extract the kinetic data and to estimate *K*_d_ values.

SDS–PAGE was performed according to Laemmli (1970 ▸). Coomassie Blue (Bradford, 1976 ▸) was used for protein determination using a commercial reagent (Bio-Rad, California, USA) with bovine serum albumin as a standard.

## 3. Results   

### 3.1. *In vitro* self-assembly of recombinantly produced chains generates the α5NC1 homohexamer   

**Figure 1:**
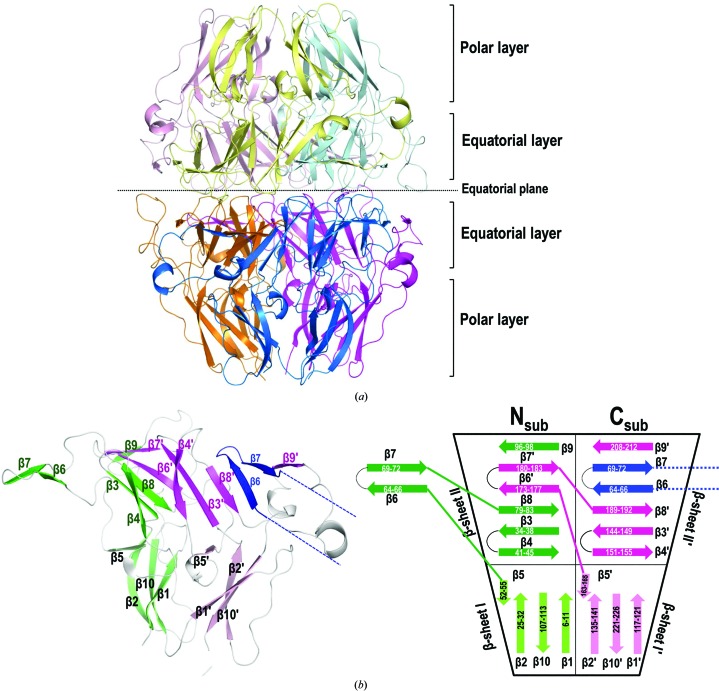
Crystal structure of α5NC1_homo_. (*a*) Cartoon representation of the crystal structure of recombinant α5NC1 formed by a hexamer composed of two trimers. Each trimer contains three subunits of α5NC1 coloured magenta/orange, cyan/pink and blue/yellow. (*b*) Cartoon representation of a subunit with a schematic representation of the N-subdomain (green) and C-subdomain (magenta) folds. The N-subdomain consists of β-sheets I (β1, β2, β5 and β10) and II (β3, β4, β6′, β7′, β8 and β9), while the C-subdomain consists of β-sheets I′ (β1′, β2′, β5′ and β10′) and II′ (β3′, β4′, β6, β7, β8′ and β9′). The scheme highlights the fact that β6 and β7 from another protomer (coloured blue) are swapped between the NC1 monomers, whereas β6′ and β7′ (coloured magenta) are swapped from the C-subdomain into the N-subdomain of the same subunit.

In an attempt to visualize the Goodpasture antigens, we tried to crystallize the α345NC1 heterohexamer by using an equimolecular mixture of pure recombinant α3NC1, α4NC1 and α5NC1 domains (Supplementary Fig. S1). We obtained crystals that diffracted X-rays to 1.85 Å resolution but that only revealed homohexamers of α5NC1 chains (Table 1 ▸ and Fig. 1 ▸ *a*), most likely representing the novel α5NC1 homohexamer recently reported in cancer cell lines (Revert *et al.*, 2018 ▸). Size-exclusion chromatography experiments confirmed a strong tendency of α5NC1 to hexamerize: ∼80% of this domain was self-organized as hexamers in solution, while isolated α3NC1 or α4NC1 eluted as monomers (Supplementary Fig. S2*a*).

The architecture of the α5NC1 homohexamer (hereafter referred to as α5NC1_homo_) is essentially the same as that of the α121NC1 hexamer obtained from natural sources (Sundara­moorthy *et al.*, 2002 ▸; Than *et al.*, 2002 ▸; Vanacore *et al.*, 2009 ▸). α5NC1_homo_ was shaped as a prolate ellipsoid of ∼92 × ∼63 Å (Figs. 1 ▸ *a* and 2 ▸, Supplementary Fig. S3*a* and Table S3). It was composed of two trimeric protomers meeting front-to-front at the equatorial plane of the ellipsoid in a nearly planar interaction surface. The subunit fold (Fig. 1 ▸ *b*) showed a tandem arrangement of two similarly folded subdomains (hereafter referred to as N-sub and C-sub), as expected for the collagen IV NC1-domain fold (Pfam PF01413; https://pfam.xfam.org/family/PF01413; Supplementary Table S4). Thus, each sub­domain of the α5NC1 subunit contains one α-helix and ten β-strands forming two antiparallel β-sheets (sheets I and II in N-sub and I′ and II′ in C-sub; a prime identifies the elements of C-sub; Fig. 1 ▸ *b*). In each protomer, the I and I′ sheets (the topologies of these sheets are β1β10β2β5 and β1′β10′β2′β5′, respectively) of the three subunits alternate, forming the polar layers of the prolate ellipsoid (the regions that link the ellipsoid to the collagenous domains at both narrow ends of the ellipsoid), whereas the equatorial layers that connect both protomers front-to-front are formed by the six alternating II and II′ sheets of the three chains (Fig. 1 ▸ *b*, left). The II and II′ sheets provide a clipping mechanism that glues the three subunits of each protomer together (Fig. 1 ▸ *b*). Each II or II′ sheet is composed of four strands of one subdomain and of two strands forming a β-hairpin projected from the corresponding sheet of the adjacent subdomain or subunit (the topologies of the II and II′ sheets are β4β3β8β6′β7′β9 and β4′β3′β8′β6β7β9′, respectively; Fig. 1 ▸ *b*). Thus, the β6′β7′ hairpin of sheet II corresponds to C-sub of the same subunit and the β6β7 hairpin of sheet II′ to N-sub of the adjacent subunit.

### 3.2. Structures of the α1NC1 homohexamer and of an unreported α3NC1 homohexamer   

**Figure 2:**
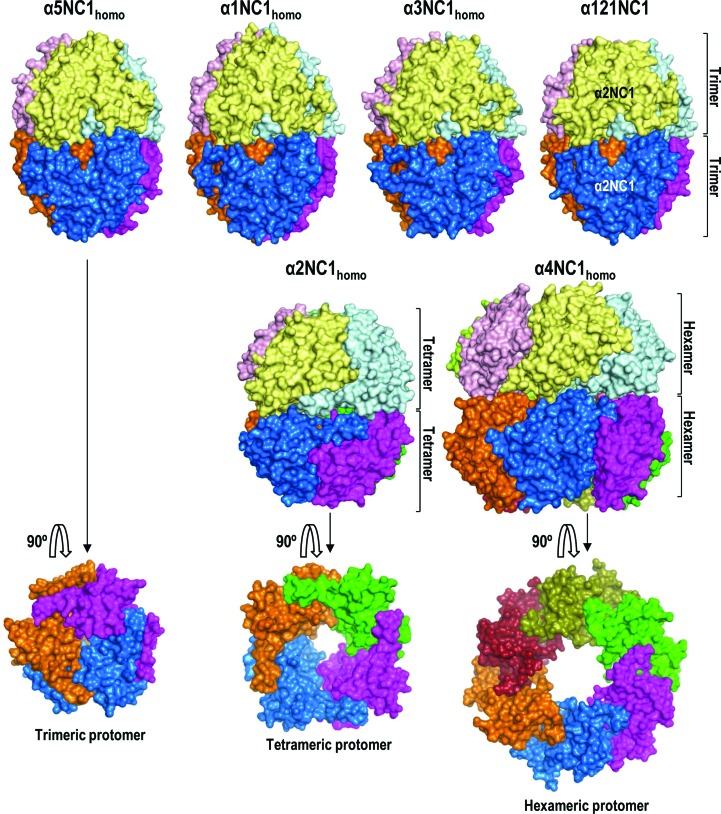
Surface representations of the quaternary structures of the canonical hexameric α5NC1_homo_, α1NC1_homo_, α3NC1_homo_ and α121NC1 and the noncanonical α2NC1_homo_ and α4NC1_homo_ organized as octamers and dodecamers, respectively. Each subunit in the assemblies is coloured differently. The top and middle rows show views in which the axis of highest molecular symmetry is vertical. In the bottom row this axis is perpendicular to the paper, allowing a view of the equatorial surface of the protomer.

*In vitro* studies have shown that α1NC1 can assemble into homohexamers (Khoshnoodi, Sigmundsson *et al.*, 2006 ▸). We grew crystals of self-assembled recombinant α1NC1 subunits (Supplementary Fig. S1) that diffracted X-rays to 1.8 Å resolution (Table 1 ▸). The asymmetric unit contained a homohexamer (α1NC1_homo_) with the same ‘canonical’ architecture as α121NC1 and α5NC1_homo_ (Fig. 2 ▸, Supplementary Fig. S3*b*). We also obtained crystals of an α3NC1 homohexamer that diffracted X-rays to 2.7 Å resolution (Table 1 ▸), revealing canonical homohexamers (α3NC1_homo_; Fig. 2 ▸, Supplementary Fig. S3*b*). Although α3NC1 homohexamers were not observed by size-exclusion chromatography (Supplementary Fig. S2*a*), plasmon resonance studies (Supplementary Fig. S2*b*) showed self-association of α3NC1 with a similar affinity as α5NC1 (*K*_d_ values of 1.71 and 4.92 µ*M*, respectively; Supplementary Fig. S2*b*) or α1NC1 (Khoshnoodi, Sigmundsson *et al.*, 2006 ▸). These self-affinities, the canonical architectures of α1NC1_homo_, α3NC1_homo_ and α5NC1_homo_, and the recent discovery of biologically formed α5NC1_homo_ (Revert *et al.*, 2018 ▸) indicate that either α1NC1_homo_ and α3NC1_homo_ hexamers exist *in vivo* and have not been discovered to date or there are biological mechanisms that prevent their formation *in vivo*.

### 3.3. Recombinant α1NC1 and α2NC1 chain mixtures reproduce the canonical naturally existing α121NC1 hexamer   

We validated the use of self-assembled recombinant NC1 domains to reflect natural structures by generating the same α121NC1 hexamer as previously isolated from BMs (Sundaramoorthy *et al.*, 2002 ▸; Than *et al.*, 2002 ▸; Vanacore *et al.*, 2009 ▸) from a 2:1 mixture of pure α1NC1 and α2NC1 chains (Supplementary Fig. S1). Crystals of recombinant α121NC1 diffracted X-rays to 2.8 Å resolution (Table 1 ▸) and had the same architecture and folding (Fig. 2 ▸, Supplementary Fig. S3*c*) as the α121NC1 hexamer isolated from BMs (the r.m.s.d. for the superimposition of all C^α^ atoms of the hexamer is 0.48 Å; Supplementary Table S3). The recombinant α121NC1 hexamer was composed of two identical protomers formed by two α1NC1 domains and one α2NC1 domain (Supplementary Figs. S4*a*, S4*b* and S4*c*). Each individual chain (designated α1NC1_α121_ and α2NC1_α121_) had an identical fold and relations to its natural counterpart (Supplementary Figs. S3*c* and S4; Supplementary Table S4; Than *et al.*, 2002 ▸; Sundaramoorthy *et al.*, 2002 ▸; Vanacore *et al.*, 2004 ▸). Thus, recombinant NC1 chains seem to be appropriate for the formation and structural characterization of collagen IV NC1-domain assemblies. Therefore, the covalent modifications and/or cross-links found in natural α121NC1 hexamers or potential directive roles of the collagenous domains or other macromolecular components do not seem to be essential for the correct self-assembly of α121NC1.

### 3.4. Structures of self-assembled α4NC1 and α2NC1 oligomers reveal possibly informative noncanonical homo-oligomers   

Given the lack of structural information on α4NC1, we grew crystals of this chain alone that diffracted X-rays to 2.8 Å resolution (Table 1 ▸). Surprisingly, they revealed a non­canonical hexameric protomer that generated a dodecamer (α4NC1_homo_) with an almost spherical shape upon application of the crystal symmetry (Fig. 2 ▸, Supplementary Fig. S5). The protomer resembled a canonical trimeric protomer, except that it had six subunits surrounding the molecular symmetry axis (Fig. 2 ▸), while the dodecamer resembled a canonical hexamer except for the increased number of sub­units, resulting in a wider equatorial circumference (∼100 Å diameter) and a widened central pole-to-pole tunnel (∼40 Å diameter *versus* ∼17 Å in canonical homohexamers) (Fig. 2 ▸, Supplementary Fig. S5).

We also crystallized and determined the structure of the isolated α2NC1 subunit at 2.5 Å resolution (Table 1 ▸). The asymmetric unit included two subunits. Following application of the crystal symmetry, each subunit formed a protomer with fourfold molecular symmetry. One of these protomers was isolated and the other generated a homo-octamer (α2NC1_homo_; Fig. 2 ▸, Supplementary Fig. S5). α2NC1_homo_ resembled a canonical hexamer, except for an increase in the number of subunits to four per protomer, enlarging the equatorial circumference somewhat (∼70 Å diameter) and widening the central tunnel (∼30 Å).

**Figure 3:**
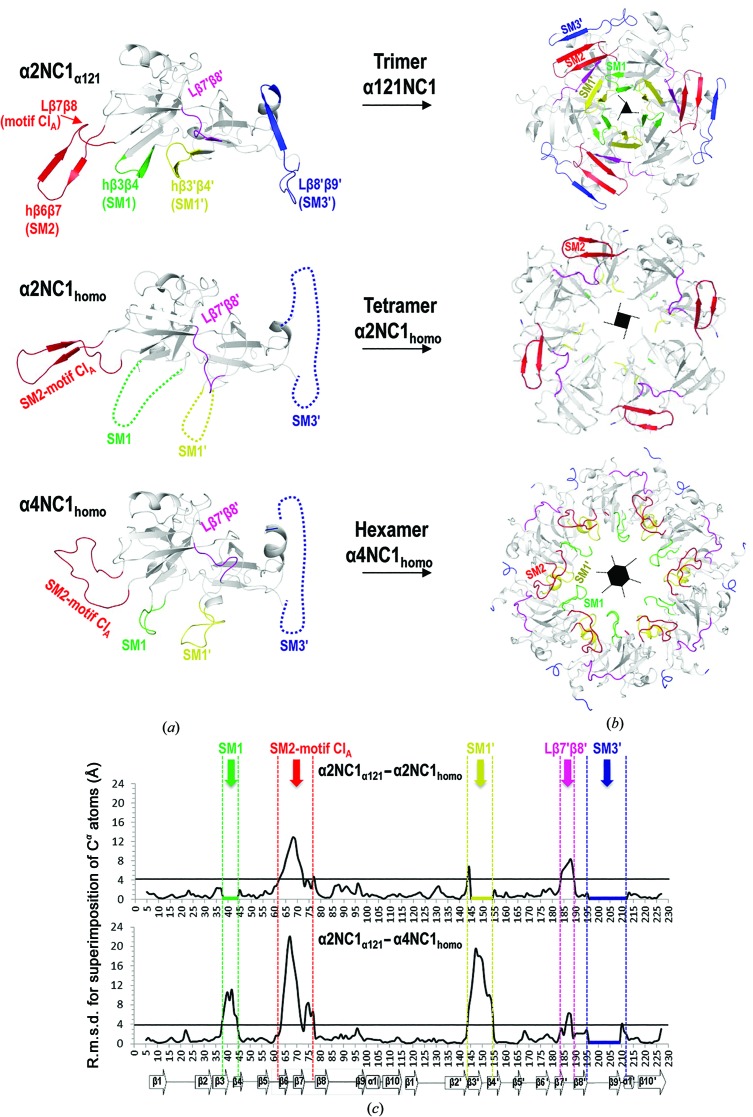
Structural comparisons of α2NC1_α121_, α2NC1_homo_ and α4NC1_homo_. The same colour scheme is used in the three panels. (*a*) Comparison of the α2NC1 and α4NC1 subunits in these oligomers, highlighting the regions with the greatest conformational difference in comparison to α2NC1_α121_. Important structural motifs (SMs; see text) are labelled and coloured. Dashed lines represent highly disordered regions that are not visible in the crystal structures. (*b*) Cartoon representation of the structures of an α121NC1 protomer and the corresponding noncanonical α2NC1_homo_ and α4NC1_homo_ tetrameric and hexameric protomers. (*c*) Plot of r.m.s.d. deviation per C^α^ atom along the sequences between α2NC1_α121_ and α2NC1_homo_ (top) and α4NC1_homo_ (bottom). Dashed vertical lines enclose the structural motifs (identified with arrows) showing high r.m.s.d.s. The thick horizontal line (residues 196–210) indicates a lack of electron density.

These two noncanonical protomers could be artifactual owing to the lack of the restrictions imposed by the triple-helical collagenous domain that is present in the complete collagen IV protomer. However, the protomers observed here in α1NC1_homo_, α3NC1_homo_, α5NC1_homo_ and α121NC1 were trimeric despite the absence of a collagenous triple helix. Therefore, the two self-assembled noncanonical structures of α2NC1 or α4NC1 subunits may lack some structural elements that are present in the canonical trimeric protomers that could intrinsically restrict the formation of protomers with more than three subunits. The comparison between the structure of the α2NC1 domain in its noncanonical homohexamer and the same domain in α121NC1 (α2NC1_α121_) revealed that there are conformational differences that might explain the greater number of subunits (Fig. 2 ▸, Supplementary Fig. S6). Similar differences were observed when α4NC1 was compared with the NC1 domains in the canonical hexamers (Fig. 3 ▸, Supplementary Fig. S6). These differences affected β-sheets II and II′ exclusively. These two sheets ensure the clipping of the protomer into a closed trimeric structure and form the equatorial layer that connects both protomers front-to-front. Here, we define three structural motifs in sheet II (Fig. 3 ▸), SM1 (hairpin β3–β4), SM2 (hairpin β6–β7) and SM3 (β9 and the preceding loop), and their homologous counterparts in sheet II′, SM1′ (hairpin β3′–β4′), SM2′ (hairpin β6′–β7′) and SM3′ (β9′ and the preceding loop) (Figs. 3 ▸ *a* and 3 ▸ *b*).

As can be seen in the canonical structures, SM1 and SM1′ (Fig. 3 ▸ *b*) face the central tunnel, creating a closed ‘barrel’-like arrangement of six alternating β4 and β4′ strands, which allows compact intertwining of three monomers (Than *et al.*, 2002 ▸). These hairpins (residues 37–45 in the case of SM1 and residues 144–154 in the case of SM1′) were not visible in the structure of α2NC1_homo_ (Fig. 3 ▸), as would be expected if they were disordered in this noncanonical homo-octamer. On the other hand, they were visible in α4NC1_homo_ but with a different conformation to that in α2NC1_α121_ (Fig. 3 ▸). These changes are expected to decrease the strength of the association of the subunits into the protomer.

As described above (Fig. 1 ▸), SM2 and SM2′ are inserted into the equatorial layers of the adjacent subunit or subdomain, respectively. In α2NC1_homo_ SM2 underwent a rigid-body rotation of 22.5° (calculated by *DYNDOM*; Hayward & Berendsen, 1998 ▸) relative to its position in α2NC1_α121_, while in α4NC1_homo_ SM2 adopted a random-coil conformation (Fig. 3 ▸, Supplementary Fig. S6). These alterations could weaken the closing belt that would have contributed to restricting the number of subunits to three in a canonical protomer.

The SM3′ motif (Figs. 3 ▸ *a* and 3 ▸ *b*) flanks SM2 peripherally (relative to the molecular symmetry axis of the protomer). SM3′ was disordered and was not visible in either α2NC1_homo_ or α4NC1_homo_ (Fig. 3 ▸), again weakening the insertion of SM2 into the II′ sheet and thus the clipping together of the oligomer. In fact, the loop connecting SM2 to β8 (Lβ7β8; residues 62–77), as well as the homologous loop connecting strands β7′ and β8′ (Lβ7′β8′; residues 185–189) in sheet II′, also adopted alternative arrangements relative to the canonical structures (Fig. 3 ▸ *c*) in α2NC1_homo_ and α4NC1_homo_. Given the closeness of the Lβ7β8 and Lβ7′β8′ loops to the SM2′ and SM2 motifs, which are inserted between strands β8 and β9 of the two homologous sheets II′ and II, these conformational changes should weaken the restricting belt that helps compel the protomer to be trimeric.

**Figure 4:**
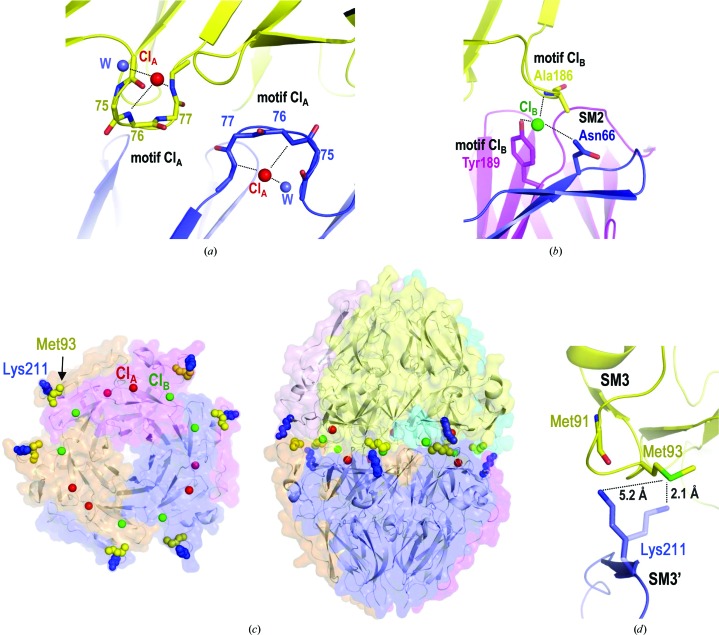
Hexamer stabilization through ion binding and potential sulfilimine-bond sites. (*a*) The chloride ion Cl_A_ (red) interacts with the main-chain amino groups of Lβ7β8 (Cl_A_ motif) and a water molecule (blue) in two opposing monomers. (*b*) The chloride ion Cl_B_ (green) binds to residues of flexible regions from three opposing monomers in the Cl_B_ motif (Ala186 in Lβ7′β8′ and Tyr189 in β8′) and Asn66 in SM2. (*c*) Semi-transparent surface representation (enclosing a cartoon representation) of the equatorial view of the protomer in the canonical α1NC1_homo_ hexamer indicating the six Cl_A_ ions (red) and six Cl_B_ ions (green) at the interface between the protomers in the hexamer. In addition, the six interfacial Met93 residues (yellow) and the six Lys211 residues (blue), three from the shown protomer and the other three from the top protomer (not shown), are also indicated to stress their proximity, which allows easy sulfilimine-bond formation. These residues and the Cl_A_ and Cl_B_ ions are also shown in the view of the complete α1NC1_homo_ hexamer illustrated on the right. (*d*) Zoom on the flexible Lβ8β9 (SM3) and β9′ (SM3′) regions of opposing monomers, highlighting the closeness of the Met93 and Lys211 residues of each region, respectively. A mere rotamer change of Lys211 would place it in position for the formation of a sulfilimine bridge with Met93.

In addition to playing a key role in the formation of the protomer, all six SMs, as well as loops Lβ7β8 and Lβ7′β8′, play crucial roles in the front-to-front interactions through the equatorial plane that glue two protomers together to generate the final particle (see the top row in Fig. 3 ▸ *b*). Loop Lβ7β8 is the chloride-binding motif (Cl_A_ motif) that assists protomer–protomer interaction across the equatorial plane following chloride binding (Cummings *et al.*, 2016 ▸; Fig. 4 ▸ *a*). The re­arrangements and structural alterations in these elements observed in α2NC1_homo_ and α4NC1_homo_ (Fig. 3 ▸) modify the equatorial plane of the tetrameric and hexameric protomers, abolishing most of the protomer–protomer interactions that are observed in the canonical hexamer. However, the persistence of some interactions through the equatorial plane can explain the association of the two noncanonical protomers of α2NC1 and α4NC1 into octamers and dodecamers, respectively, which overall follow the general plan of the canonical hexameric assembly. The presence of an isolated tetrameric protomer that is not associated into an octamer in the α2NC1_homo_ crystal is structural evidence of weakened interactions across the equatorial plane.

**Figure 5:**
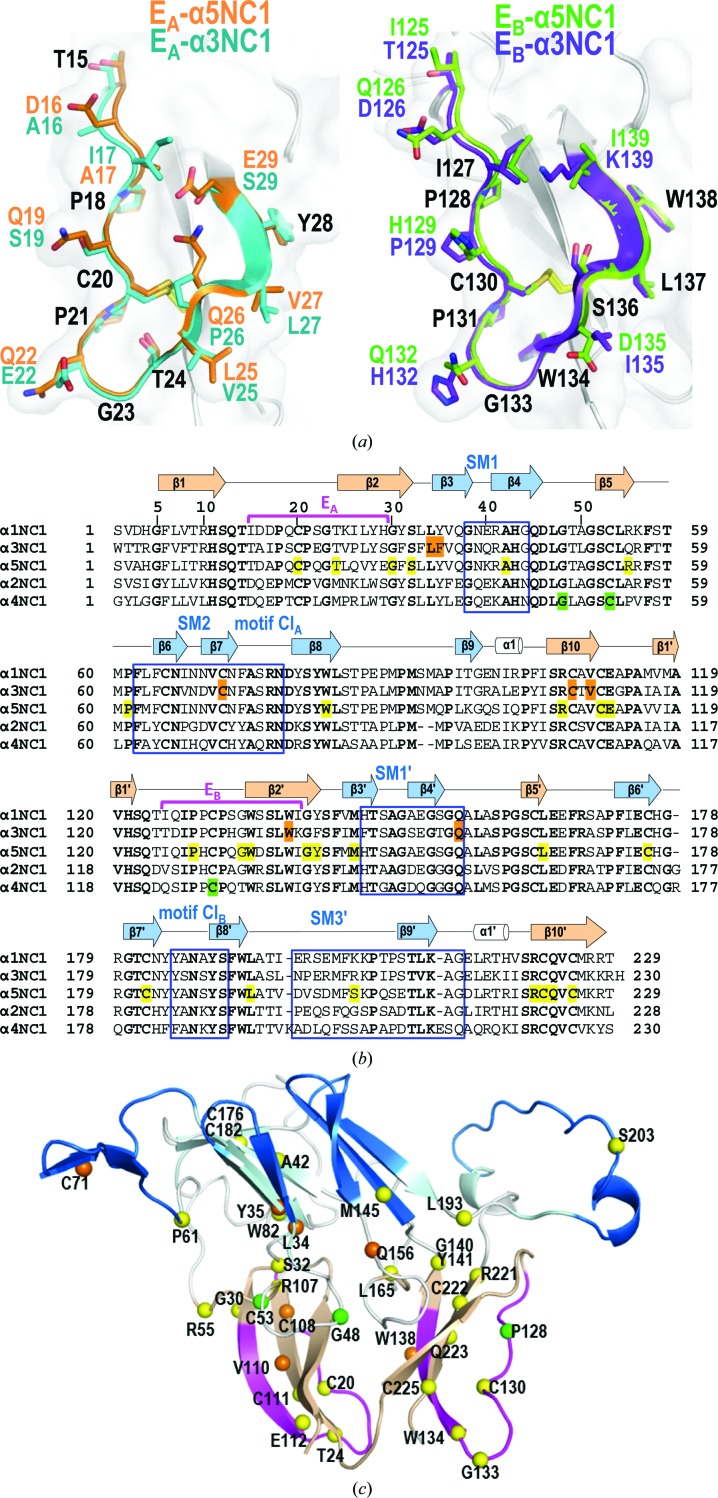
Structural bases of Alport’s and Goodpasture’s syndromes. (*a*) Goodpasture’s epitopes E_A_ (left) and E_B_ (right) as seen in the superimposed structures of subunits of α3NC1_homo_ (cyan or blue) and α5NC1_homo_ (orange or green). The same colour code is used for the amino-acid side chains (except for invariant residues, which are shown in black). (*b*) Structure-assisted sequence alignment of the α1–5NC1 chains. Arrows indicate β-strands and cylinders indicate α-helices. β-Strands of the I/I′ and II/II′ sheets are coloured light pink and light blue, respectively. Blue rectangles enclose flexible regions and Cl motifs (labelled), horizontal magenta lines mark the E_A_ and E_B_ epitopes (also labelled) and coloured shadowing indicates the residues reported to host missense mutations in Alport’s syndrome. (*c*) Alport’s syndrome missense mutations (listed in Supplementary Table S5) are mapped onto the structure of an α5NC1 subunit. Yellow spheres represent mutations in this chain, while superimposed mutations of α3NC1 and α4NC1 are coloured orange and green. β-Sheets I/I′ and II/II′ are coloured light pink and light blue, respectively, E_A_ and E_B_ are shown in magenta and flexible SM regions and Cl motifs are presented in deep blue.

Overall, a comparison of noncanonical and canonical oligomers (Figs. 3 ▸ *a* and 3 ▸ *b*) revealed that the SM1, SM1′ and SM2 flexible regions are key determinants in protomer formation, while the loops connecting these motifs, Lβ7β8 (motif Cl_A_), Lβ7′β8′ and SM3′, are needed to establish the protomer–protomer interactions that generate the hexamer. It is interesting that among these structurally more variable regions (Fig. 3 ▸ *c*), the loops that connect β8 and β9 (Lβ8β9), β6′ and β7′ (Lβ6′β7′), and β8′ and β9′ (Lβ8′β9′) present length differences of one or two residues among the various types of NC1 chains (see below; Fig. 5 ▸ *b*), suggesting that these regions provide structural variability to the oligomers. Indeed, previous work with the natural α121NC1 heterohexamer has reported structural inter-chain variability in the conformations of these loops (Sundaramoorthy *et al.*, 2002 ▸; Than *et al.*, 2002 ▸). Here, these loops show variations in the structures of our canonical homohexamers (Supplementary Table S4 and Supplementary Fig. S7).

In summary, mobility and conformational changes are concentrated in the equatorial layer formed by β-sheets II and II′, a layer that is crucial for clipping the structure of the protomer and that connects both protomers front-to-front, whereas sheets I and I′ of the polar layer exhibit high structural conservation in canonical and noncanonical protomers (Fig. 3 ▸ *c*). In the polar layer only the loop between β1 and β2 exhibited substantial movement (translated into higher values for the r.m.s.d. between odd-numbered and even-numbered NC1 chains; Supplementary Fig. S7), caused by a short flapping movement (∼30°) with no alteration of the actual structure of this loop, leading only to changes in its position. Given their high structural stability, sheets I and I′ may form the initial structural core of the subunit, folding first, while the flexible regions of β-sheets II and II′ could be involved in the termination of the folding process, ensuring proper protomer assembly. At this point, we are unable to identify the reasons for the insertion of just one extra subunit or of three extra subunits in the α2NC1 and α4NC1 protomers, respectively. However, it seems clear that the disorder or loss of the SM1 and SM1′ secondary structure observed in the noncanonical α2NC1_homo_ and α4NC1_homo_ protomers, which leads to loss of the β-barrel-like organization around the central tunnel, could affect SM2 and the Cl_A_ motif of the same subunit, resulting in the displacement of these two elements away from the central tunnel. The structural disruption of SM1′ could also affect the adjacent β8′ strand (which loses its secondary structure in the noncanonical protomers) and the insertion of SM2 in the next subunit (Fig. 3 ▸). SM2 is a key protomer-clipping element that would become relaxed in the two noncanonical protomers, allowing the insertion of extra subunits. In turn, the resulting abnormal placement of SM2 could prevent interactions with SM3′, which would avoid the generation of a narrow canonical protomer.

### 3.5. Chloride-mediated protomer–protomer stabilization and potential for sulfilimine cross-linking in canonical but not in noncanonical self-assembled oligomers of recombinant NC1 chains   

The α121NC1 structures obtained from natural sources showed the presence of ions and covalent sulfilimine bonds associated with stabilization of the hexamers (Vanacore *et al.*, 2004 ▸; Than *et al.*, 2002 ▸; Robertson *et al.*, 2014 ▸). Chloride ions were also present in our α1NC1_homo_, α3NC1_homo_ and α5NC1_homo_ structures at two different positions in the protomer–protomer interface. Six chloride ions (Cl_A_), one per monomer, were modelled at the position previously observed in the natural α121NC1 structure (Figs. 4 ▸ *a* and 4 ▸ *c*). Cl_A_ interacted with the amino groups of the Cl_A_ motif. In the structure of the α121NC1 hexamer obtained from the BM of bovine lens capsule (Cummings *et al.*, 2016 ▸), this motif was one of the flexible regions shown to be crucial for triggering the assembly of the two α121NC1 protomers into a hexamer.

Six additional chloride ions (Cl_B_), again one per monomer, were also observed in the α1NC1_homo_, α3NC1_homo_, α5NC1_homo_ and α121NC1 hexamers (Figs. 4 ▸ *b* and 4 ▸ *c*). In the α121NC1 structure from the BM of bovine lens the nonprotein electron densities at these positions were interpreted as potassium ions, while in the equivalent α121NC1 structures from the BMs of bovine and human placenta they were modelled as bromide ions and acetate, respectively (Vanacore *et al.*, 2004 ▸). We modelled the densities at these positions as Cl^−^ ions since the nature and geometry of the contacts mediated by them (with a hydroxyl group and with amino groups) and the electron densities fitted best with the presence of this anion, which is abundant in the crystallization drop. Cl_B_ was located at the interprotomer interface and was involved in an interaction network between conserved residues that are present in Lβ7′β8′ (hereafter referred to as the Cl_B_ motif) and SM2 from three different monomers of opposing protomers (Fig. 4 ▸ *c*). In contrast, in the noncanonical homoligomers α2NC1_homo_ and α4NC1_homo_ all chloride ions were missing mainly owing to the rearrangement of the Cl_A_ and Cl_B_ motifs as well as of SM2 and β8′ (Fig. 3 ▸), which prevented the coordination of these chlorides. Since all recombinant proteins used were prepared in NaCl buffers, and since hexamer assembly in the BM from bovine lens has recently been demonstrated to be chloride-dependent (Cummings *et al.*, 2016 ▸), the conformational changes observed in α2NC1_homo_ and α4NC1_homo_ that prevent chloride binding may hinder the final protomer–protomer assembly.

As expected, in the final stage of network assembly the natural NC1 hexamers of collagen IV cross-linked by covalent sulfilimine bonds between Met93 from SM3 of one monomer and Lys211 from SM3′ of another monomer facing across the equatorial plane were formed (α1NC1 residue numbering; Than *et al.*, 2002 ▸; Vanacore *et al.*, 2009 ▸). The formation of the sulfilimine bonds is catalyzed by peroxidasin, an enzyme found in BMs (Bhave *et al.*, 2012 ▸). No sulfilimine bonds were seen in the structures presented in this work, possibly because all of the proteins were produced in a recombinant system in which either the conditions were inadequate for the catalytic activity of peroxidasin or this enzyme was absent. However, in the α1NC1_homo_, α3NC1_homo_, α5NC1_homo_ and α121NC1 structures Lys211 interacts *via* a hydrogen bond with the SM3 containing Met93 of an opposing monomer (Figs. 4 ▸ *c* and 4 ▸ *d*). An alternative conformation of the Lys211 side chain could allow sulfilimine-bond formation with Met93 (Fig. 4 ▸ *d*). This was not the case for α2NC1_homo_ and α4NC1_homo_ (Fig. 3 ▸), where the inappropriate location of these residues for sulfilimine-bond formation reflects the high flexibility of β9′ in SM3′, strongly suggesting that the structural elements involved in protomer–protomer assembly acquire their final conformation at the end the NC1 folding process. Thus, NC1 folding is correlated with the proposed monomer–protomer–hexamer sequential assembly model for collagen IV network formation (Boutaud *et al.*, 2000 ▸; Kalluri, 2003 ▸; Khoshnoodi, Cartailler *et al.*, 2006 ▸; Cummings *et al.*, 2016 ▸).

### 3.6. Insight into Alport’s and Goodpasture’s syndromes   

**Figure 6:**
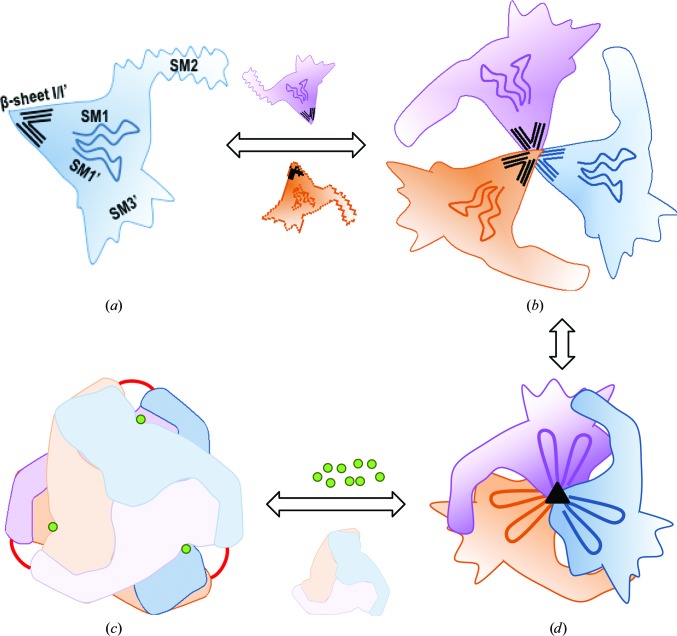
Proposed model for canonical hexamer assembly. Individual monomers (*a*) start to nucleate a protomer *via* β-sheets I/I′ (*b*). Next, the SM1/1′ and SM2/SM2′ flexible regions from β-sheets II/II′ are stabilized in the nascent protomer, resulting in favoured additional intersubunit interactions within the protomer (*c*). Final stabilization is attained with the proper folding of SM3′ and of the Cl_A_ and Cl_B_ motifs that allow the binding of chloride ions (green spheres) (*d*). The two protomers in the hexamer are now ready to be joined by sulfilimine bonds (red lines).

The α345 collagen IV network is a major component of the glomerular BM and its alteration underlies the pathogenesis of Alport’s and Goodpasture’s syndromes (Hudson *et al.*, 2003 ▸; Pedchenko *et al.*, 2010 ▸). In Alport’s syndrome, mutations in these chains, including α5NC1, lead to a defective network assembly, causing the multi-laminar splitting of the glomerular BM. In Goodpasture’s disease autoantibodies directed to the α3NC1 and α5NC1 domains are generated. Our structures of α3NC1_homo_, α4NC1_homo_ and α5NC1_homo_ show that two structurally equivalent regions of α3NC1, E_A_ and E_B_, as well as the homologous regions of α5NC1, encompass the Goodpasture epitopes (Figs. 5 ▸ *a* and 5 ▸ *b*; Cui *et al.*, 2016 ▸). Furthermore, the Alport missense mutations in the NC1 domains (Crockett *et al.*, 2010 ▸; see Supplementary Table S5) predominantly map to the more structurally stable regions of the NC1 domain (∼90% of the mutations), mainly β-sheets I/I′, with few mutations mapping to those regions that showed high flexibility in α2NC1_homo_ and α4NC1_homo_ (∼10% of the mutations; Figs. 5 ▸ *b* and 5 ▸ *c*). Since the structural core of the NC1 domains should acquire its conformation in the initial steps of NC1 folding (see Fig. 6 ▸ and *Discussion* [Sec sec4]) to nucleate protomer assembly, these mutations could strongly compromise collagen IV network formation. Supporting this idea, most of the Alport mutations (68%) are found in residues with high structural relevance, such as cysteine, glycine or proline (Supplementary Table S5).

To our knowledge, the structures presented here represent the first visualization of the Goodpasture autoantigen. The regions that encompass the E_A_ and E_B_ epitopes lie in β-sheets I/I′, a more structurally constant and fixed part of the NC1 structural core (Figs. 1 ▸ *b* and 5 ▸ *c*). The comparison of E_A_ and E_B_ of α3NC1 and α5NC1 also showed that they are identical in structure and also to the corresponding part of α1NC1, although no antigenicity has been determined in the latter chain in patients with Goodpasture’s disease (Supplementary Figs. S8 and S9). Solvent-accessibility analysis of residues at the E_A_ and E_B_ epitopes in the α3NC1 and α5NC1 structures, in comparison to the corresponding residues in α1NC1, showed similar exposed surface areas in either monomers or hexamers. Very small differences in solvent accessibility were observed for Leu27 and Tyr28 in E_A_ and for Pro131 and Trp134 in E_B_ (Supplementary Fig. S10). These residues are highly conserved among NC1 chains (Supplementary Figs. S8*a* and S9*a*). They form a hydrophobic patch on the exposed surface of the protomer (Supplementary Fig. S11*a*). Further comparisons with the corresponding regions of α2NC1_homo_ and α4NC1_homo_ revealed conservation of the fold in this region even in these noncanonical oligomers (Supplementary Figs. S11*b* and S11*c*). Therefore, the differential antigenicity observed between chains must be owing to sequence determinants, as has recently been proposed (Cui *et al.*, 2016 ▸).

## 4. Discussion   

The very recent report of the existence of the α5NC1 homohexamer *in vivo* (Revert *et al.*, 2018 ▸) gives credence to the view that the canonical α1NC1 and α3NC1 homohexamers presented here may also exist *in vivo*, particularly given their structural stability, their proper binding of chloride ions for hexamer stabilization and their correct disposition of the residues that mediate the sulfilimine cross-linking bonds that stabilize the hexamer. Their spontaneous self-assembly in solutions of individual components, and even in mixtures of different components, attests to their thermodynamic stability and the absence of important kinetic barriers against their formation.

The finding that only a few combinations of collagen IV chain types are present in BMs, among the >1000 possible combinations of the six collagen IV chains, is difficult to explain based only on spontaneous thermodynamically driven assembly. Although the contribution of the collagenous parts to chain selection has not yet been clarified, our findings with isolated NC1 domains without the collagenous parts suggest the existence of regulatory mechanisms that could allow or guide the selection of a given chain combination. Spontaneous assembly has been proposed for α121NC1, where the α2NC1 chain shows a higher affinity to interact with α1NC1 than with itself (Khoshnoodi, Sigmundsson *et al.*, 2006 ▸). In fact, we show here that 2:1 mixtures of α1NC1 and α2NC1 chains spontaneously generate the natural α121NC1 heterohexamer. Conversely, our experimental results have shown that equimolecular mixtures of α3NC1, α4NC1 and α5NC1 only produce α5NC1_homo_ crystals. Therefore, further studies will be required to clarify the mechanism underlying the alternative formation of α5 and α345 networks *in vivo*.

Our findings of noncanonical assemblies for α2NC1 and α4NC1 homo-oligomers and the structural changes observed in the chains forming these noncanonical protomers in comparison to α2NC1_α121_ and to the chains in α1NC1_homo_, α3NC1_homo_ and α5NC1_homo_ could imply that chain folding is closely related to protomer assembly. It is unlikely that α2NC1_homo_ and α4NC1_homo_ could represent stable physio­logical assemblies, but the structures of the individual chains in these noncanonical oligomers might provide a frozen glimpse of transient conformational states in the process of NC1 folding and hexamer building. This is supported by the identical folding of many parts of these non­canonical oligomers and canonical structures, particularly in polar layers (β-sheets I and I′), and the restriction of flexible regions in both noncanonical structures to essentially the same sequence stretches belonging to the equatorial layer (mainly β-sheets II and II′).

As already mentioned, β-sheets I/I′ could act as a stable structural core nucleating the NC1 domains, which could prevent their final folding until integrated into a trimer, since the SM2 would have to be inserted into β-sheet II′ of an adjacent subunit. A plausible scenario for NC1 equatorial layer folding that is compatible with our observations with non­canonical oligomers would involve first the formation of the central β-barrel-like structure by SM1/SM1′, with the immediate insertion of SM2′ into the forming β-sheet II of the same subunit. This would make SM2 ready for insertion into the SM3′ of an adjacent folding protomer. Following this insertion, β-sheet II′ would acquire its final structure. The process of a central β-barrel-like organization reduces the size of the central tunnel, restricting the number of monomers in the protomer to three, which is further stabilized by the presence of the triple-helical collagenous domain. Subsequently, flexible loops (Cl_A_ and Cl_B_ motifs) at the equatorial plane of the protomer could acquire a competent conformation for chloride binding, leading to the final assembly of the hexamer involving the interaction of two protomers through their flat surfaces in a chloride-rich medium, *i.e.* the extracellular fluid, with further hexamer stabilization by sulfilimine cross-linkings (Fig. 6 ▸). In the previous description, the potential participation of an uncharacterized cellular machinery in assisting this process of chain sorting, folding and assembly has not been taken into account. It has recently been reported that the production of an α5 network in cancer cell lines is dependent on the expression and activity of the Goodpasture antigen-binding protein (GPBP). This strongly suggests that this machinery exists and that GPBP forms an important part of it (Revert *et al.*, 2018 ▸).

Our present work helps to clarify why missense mutations affecting NC1 domains in Alport’s syndrome are more frequently found in the structural core of the NC1 domain. Given the key role of β-sheets I/I′ in subunit folding that is suggested by our findings, mutations in this region are likely to have increased visibility (‘eloquent mutations’) since they will have a higher impact on the final structure in comparison to mutations in structurally less relevant parts. Thus, the genetic variants of Alport’s syndrome owing to missense mutations in this domain can be considered to cause a folding disease, as is supported by the occurrence of the unfolded protein response and of endoplasmic reticulum stress in Alport’s syndrome (Gould *et al.*, 2007 ▸; Pieri *et al.*, 2014 ▸). With this in mind, chemical chaperones could be used to help to restore the proper conformation and folding in cases with certain missense mutants (Murray *et al.*, 2014 ▸; Wang *et al.*, 2017 ▸).

In Goodpasture’s autoimmune disease, the autoepitopes are immunoreactive only when the native α345NC1 hexamers are dissociated into monomers or dimer subunits (Hudson *et al.*, 2003 ▸; Pedchenko *et al.*, 2010 ▸). The underlying mechanism has been attributed to conformational changes forming epitopes in dissociated α3 and α5 subunits that bind the pathogenic autoantibodies (Calvete *et al.*, 2006 ▸; Pedchenko *et al.*, 2010 ▸). Our crystal structures of the α3NC1 and α5NC1 homohexamers provide the first view of the arrangement of the E_A_ and E_B_ regions, which are known critical components of two epitopes that reside in the dissociated α3 and α5 subunits of native α345NC1 hexamers. In the homohexamers these regions are exposed on the surface and are presumably unreactive to antibody binding, as they are in the native α345NC1 hexamers. This knowledge of E_A_ and E_B_ structures will be critical in elucidating the structural mechanism that enables epitope presentation only in dissociated subunits. Potentially, the mechanism will shed light on the aetiology of Goodpasture’s disease.

It is conceivable that the extreme structural stability of the regions visualized here as pertaining to the E_A_ and E_B_ epitopes could be related to a high antigenicity because of the presentation of a single conformer, while other areas of the protein with higher flexibility could present many different conformations, populated with reduced frequencies, to the antibody-making machinery. The preference of Goodpasture antibodies to recognize the α3NC1 chain could be explained by its sequence composition, although the recent description of Goodpasture’s disease with autoantibodies reactive exclusively to α5NC1 (Cui *et al.*, 2016 ▸) opens the door to additional factors being implicated in pathogenesis. Anti-α3NC1 autoantibodies are found in most Goodpasture’s disease patients. The presence of α5NC1-specific autoantibodies could be considered to be to some extent atypical or exceptional in the context of Goodpasture’s disease, and thus α3NC1 must exhibit some singularity that makes it more susceptible to undergoing autoimmune attack in comparison with other chains.

## Supplementary Material

PDB reference: collagen type IV α1α2α1NC1, 5nax

PDB reference: α1NC1, 5nay

PDB reference: α2NC1, 5nb2

PDB reference: α3NC1, 5nb0

PDB reference: α4NC1, 5nb1

PDB reference: α5NC1, 5naz

Supplementary Tables and Figures.. DOI: 10.1107/S2052252518012459/tj5018sup1.pdf

## Figures and Tables

**Table 1:** Crystallographic data and refinement statistics for crystals of recombinant NC1 chains and their mixtures Values in parentheses are for the highest resolution shell.

	α1NC1_homo_	α2NC1_homo_	α3NC1_homo_	α4NC1_homo_	α5NC1_homo_	α121NC1
Crystallization						
Crystallization mixture	1.4 *M* Li_2_SO_4_, 0.1 *M* MES pH 6.5	22% polyvinylpyrrolidone K15, 0.1 *M* Na_2_SO_4_, 0.1 *M* MES pH 6.5	16% PEG 3350, 0.2 *M* MgCl_2_, 0.1 *M* bis-tris propane pH 7.5	6% PEG 3350, 0.2 *M* Na acetate, 0.1 *M* MES pH 6.5	20% PEG 8000, 0.2 *M* NaCl, 0.1 *M* CAPS pH 10.5	10% PEG 8000, 0.2 *M* Mg acetate
Additions for crystal harvesting	15% sucrose, 7.5% ethylene glycol	None	PEG 3350 increased to 40%	Two-step graded increase to 40% PEG 3350	PEG 8000 increased to 40%	PEG 8000 increased to 20% and 20% sucrose added
Data collection						
Light source	ID23-2, ESRF	BL13, ALBA	ID23-1, ESRF	ID23-1, ESRF	ID14-1, ESRF	ID29, ESRF
Wavelength (Å)	0.87	0.98	1.00	1.00	0.98	1.25
Space group	*P*2_1_2_1_2_1_	*I*422	*H*3	*C*222_1_	*P*4_1_32	*P*3_1_21
*a*, *b*, *c* (Å)	94.9, 127.1, 130.5	94.3, 94.3, 223	131.5, 131.5, 248.9	145.6, 167.6, 155.4	121.3, 121.3, 121.3	126.2, 126.2, 216.2
α, β, γ (°)	90, 90, 90	90, 90, 90	90, 90, 120	90, 90, 90	90, 90, 90	90, 90, 120
Resolution (Å)	57.12–1.80 (1.90–1.80)	112.81–2.50 (2.64–2.50)	65.76–2.70 (2.85–2.70)	49.44–2.80 (2.95–2.80)	60.63–1.85 (1.95–1.85)	48.75–2.82 (2.97–2.82)
*R*_merge_ † (%)	7.6 (38.8)	13.4 (168.8)	8.4 (26.4)	7.9 (33.6)	10.0 (35.4)	9.2 (36.7)
*R*_p.i.m._ † (%)	4.8 (24.8)	2.7 (33.3)	6.9 (22.3)	3.2 (13.1)	4.9 (17.4)	4.0 (16.6)
Mean *I*/σ(*I*)	10.7 (2.8)	24.1 (2.4)	11.2 (4.9)	14.6 (5.6)	12.1 (4.1)	12.7 (3.6)
Completeness (%)	98.2 (99.0)	100 (100)	98.4 (95.0)	99.9 (100.0)	99.3 (99.9)	98.9 (99.8)
Multiplicity	3.4 (3.3)	25.2 (26.4)	2.2 (2.1)	7.3 (7.5)	4.8 (4.8)	5.4 (5.3)
Refinement						
Resolution (Å)	57.12–1.80	112.81–2.50	65.76–2.70	49.44–2.80	60.63–1.85	48.75–2.82
No. of reflections						
Total/unique	479345/142960	456608/18124	96546/43361	341405/46979	125648/26393	258124/48121
Unique	68684/20928	68350/2593	12599/6111	51123/6783	18482/3812	36709/6976
*R*_work_/*R*_free_ (%)	17.6/19.9	24.6/27.9	16.3/17.6	23.2/28.0	18.5/19.0	14.1/18.4
Protein chains	6	2	8	6	1	6
No. of atoms						
Protein	10612	2739	13891	9603	1764	10413
Ligands/ions	52	0	16	0	25	11
Water	789	55	232	67	116	141
*B* factors (Å^2^)						
Protein	18.5	63.7	26.6	56.9	12.9	65.5
Ligands/ions	24.6	0	27.1	0	20.7	65.3
Water	26.7	52.9	31.2	47.6	18.8	50.9
R.m.s. deviations						
Bond lengths (Å)	0.009	0.008	0.008	0.009	0.009	0.009
Bond angles (°)	1.27	1.34	1.15	1.33	1.25	1.19

† A single crystal was used for each structure.
