# Structures of collagen IV globular domains: insight into associated pathologies, folding and network assembly. Corrigendum

**DOI:** 10.1107/S2052252520007216

**Published:** 2020-06-13

**Authors:** Patricia Casino, Roberto Gozalbo-Rovira, Jesús Rodríguez-Díaz, Surajit Banerjee, Ariel Boutaud, Vicente Rubio, Billy G. Hudson, Juan Saus, Javier Cervera, Alberto Marina

**Affiliations:** a Department of Biochemistry and Molecular Biology/ERI BIOTECMED, Universitat de València, Dr Moliner 50, Burjassot, 46100 Valencia, Spain; b Instituto de Biomedicina de Valencia, Consejo Superior de Investigaciones Científicas (IBV–CSIC), Jaume Roig 11, 46010 Valencia, Spain; c CIBER de Enfermedades Raras (CIBERER–ISCIII), Spain; d Laboratorio de Reconocimiento Molecular, Centro de Investigación Príncipe Felipe, Eduardo Primo Yúfera 3, 46012 Valencia, Spain; e Departamento de Microbiología, Facultad de Medicina at Universitat de València, Blasco Ibáñez 17, 46010 Valencia, Spain; f Department of Defense, Center for Prostate Disease Research, Bethesda, Maryland, USA; g BioStratum Inc., Durham, North Carolina, USA; h Department of Medicine at Division of Nephrology and Hypertension, Vanderbilt University Medical Center, Nashville, TN 37232, USA; i Center for Matrix Biology, Vanderbilt University Medical Center, Nashville, TN 37232, USA; j Department of Biochemistry, Vanderbilt University Medical Center, Nashville, TN 37232, USA; k Department of Pathology, Microbiology and Immunology, Vanderbilt University Medical Center, Nashville, TN 37232, USA; l Department of Cell and Developmental Biology, Vanderbilt University Medical Center, Nashville, TN 37232, USA; m Vanderbilt Ingram Cancer Center, Vanderbilt University Medical Center, Nashville, TN 37232, USA; n Vanderbilt Institute of Chemical Biology, Vanderbilt University Medical Center, Nashville, TN 37232, USA; o Departamento de Bioquímica y Biología Molecular at Facultad de Medicina y Odontología, Universitat de València, Blasco Ibáñez 15-17, 46010 Valencia, Spain

**Keywords:** collagen type IV, network assembly, (IV)NC1 hexamers, Goodpasture’s disease, Alport’s syndrome

## Abstract

The article by Casino *et al.* [*IUCrJ* (2018). **5**, 765–779] is corrected.

## Abstract 2

Corrigendum to the article by Casino *et al.* [*IUCrJ* (2018). **5**, 765–779].

The following corrections to the article by Casino *et al.* (2018 ▸) are given.

(*a*) The name of one of the contributing authors, Surajit Banerjee, was incorrectly listed as Sreedatta Banerjee.

(*b*) The address of the author Surajit Banerjee, when the article was published, should have been Northeastern Collaborative Access Team and Department of Chemistry and Chemical Biology, Cornell University, Argonne, Illinois, USA.
