# Supplementary material for: Structures of collagen IV globular domains: insight into associated pathologies, folding and network assembly
Source: IUCrJ. 2018 Oct 10;5(Pt 6):765–79. doi: 10.1107/S2052252518012459 (PMC6211539; doi:10.1107/S2052252518012459)
Supplement: Supplementary file 1 [file m-05-00765-sup1.pdf]

IUCrJ

Volume 5 (2018)

Supporting information for article:

Structures of collagen IV globular domains: insight into associated pathologies, folding and network assembly

Patricia Casino, Roberto Gozalbo-Rovira, Jesús Rodríguez-Díaz, Sreedatta Banerjee, Ariel Boutaud, Vicente Rubio, Billy G. Hudson, Juan Saus, Javier Cervera and Alberto Marina

Table S1 Primers used for αNC1 amplification.

Product	Primer name	Primer sequences
α1(IV)NC1	NCI1_Flag1/2_FWD	GACAAGGGCCCCGATGGGTTGCCAGGATCCATGGGGCCC
	NCI1_Flag_FWD	GACTACAAGGACGACGATGACAAGGGCCCCGATGGGTTGCCAGGATCC
	NCI1_REV	TAGCTGAGTCAGGCTTCATTATGTTCT
	NCI1_KpnI_REV	CCATGTTGGTACCTTAGCTGAGTCAGGCTTCATTATGTTCT
α2(IV)NC1	NCI2_Flag1/2_FWD	GACAAGGGCCGTCCAGGGAGCCCGGGCCTGCCGGGTATG
	NCI2_Flag_FWD	GACTACAAGGACGACGATGACAAGGGCCGTCCAGGGAGC
	NCI2_REV	TTCCTGGCACGCGCCGGCTCACAGGTT
	NCI2_SacI_REV	TTCCTGGCAGAGCTCGGCTCACAGGTT
α4(IV)NC1	NCI4_Flag1/2_FWD	GACAAGCTAGCTGGTCCCATTGGGGATCCTGGGCCCAAA
	NCI4_Flag_FWD	GACTACAAGGACGACGATGACAAGCTAGCTGGTCCCATTGGGGATCCTGGGCCC
	NCI4_REV	GCCGAGGGCCCCTAGCTATACTTCACGCAG
	NCI4_KpnI_REV	ATTGGTACCGCCGAGGGCCCCTAGCTA
α5(IV)NC1	NCI5_Flag1/2_FWD	GACAAGGGTCCAGATGGATTGCAAGGTCCCCCAGGTCCC
	NCI5_Flag_FWD	GACTACAAGGACGACGATGACAAGGGTCCAGATGGATTGCAAGGTCCC
	NCI5_Flag_REV	AAGGAATTCTTCAAAATGTTATGTCCT
	NCI5_SacI_REV	GGTGAGCTCAAGGAATTCTTCAAAATG
Cloning Bm40 and Flag	Flag_SignalP1_FWD	AGGGCTCTGGCAGCCCCACTAGCCGACTACAAGGACGACGATGACAAG
	SignalP1_SignalP2_FWD	TTCTTTCTCCTTTGCCTGGCCGGGAGGGCTCTGGCAGCCCCACTAGCC
	SignalP2_BamHI_FWD	AGGGGGATCCATGAGGGCCTGGATCTTCTTTCTCCTTTGCCTG
	SignalP2_XhoI_FWD	AGGGCTCGAGATGAGGGCCTGGATCTTCTTTCTCCTTTGCCTG

Table S2 Residue numbers for the structures of α1, α2, α3, α4 and α5 NC1 domains and their corresponding residue numbers according to the UniprotKB accession number.

Collagen α(IV) NC1 domain	Residue numbers in each chain	Corresponding residue numbers in the complete α(IV) chain	
		UniprotKB accession number	Residue number
α1NC1	1-229	P02462	1441-1669
α2NC1	1-228	P08572	1485-1712
α3NC1	1-230	Q01955	1441-1671
α4NC1	1-230	P53420	1461-1690
α5NC1	1-229	P29400	1461-1685

Table S3 Root mean square deviations (r.m.s.d.), in Å, for the superposition of the structures of NC1 hexamers.

The number of Cα atoms superimposed is indicated between parentheses.

Rmsd (Å)	α1NC1 $_{homo}$	α3NC1 $_{homo}$	α5NC1 $_{homo}$	α121NC1	α121NC1(PDB:1LI1)*
**α1NC1 $_{homo}$**	-	0.62 (1342)	0.55 (1356)	0.59 (1335)	0.73 (1346)
**α3NC1 $_{homo}$**		-	0.67 (1344)	0.72 (1327)	0.84 (1329)
**α5NC1 $_{homo}$**			-	0.67 (1338)	0.70 (1338)
**α121NC1**				-	0.48 (1348)

*Human α121NC1 obtained from placenta, PDB file1LI1.

Table S4 Root mean square deviations (r.m.s.d.), in Å, for the superposition of the structures of the various recombinant NC1 chains crystallized here and the α1-2NC1 chains of human placenta.

The number of Cα atoms superimposed is indicated between parentheses.

Rmsd (Å)	α1NC1$_{α121}$	α2NC1*	α2NC1$_{α121}$	α2NC1$_{homo}$	α3NC1$_{homo}$	α4NC1$_{homo}$	α5NC1$_{homo}$
**α1NC1***	0.35 (226)				0.65 (224)		0.47 (226)
**α1NC1$_{α121}$**	-		0.75 (218)	1.44 (166)	0.53 (225)	1.27 (178)	0.41 (226)
**α2NC1***		-	0.45 (224)	1.20 (169)		1.27 (174)	
**α2NC1$_{α121}$**	0.84 (219)	0.45(224)		1.22 (168)	0.88 (219)	1.25 (175)	0.79 (219)
**α1NC1$_{homo}$**	0.36 (226)				0.53(225)		0.41 (226)
**α2NC1$_{homo}$**		-	-	-	1.45 (167)	1.40 (164)	1.46 (167)
**α3NC1 $_{homo}$**					-	1.26 (179)	0.58 (225)
**α4NC1$_{homo}$**						-	1.23 (178)
**α5NC1$_{homo}$**							-

*From human placenta, PDB file 1LI1.

Table S5 Missense mutations found in Alport's syndrome patients in α5NC1, α3NC1 and α4NC1. α5NC1 mutations have been extracted from The Alport syndrome COL4A5 variant database (www.arup.utah.edu/database/ALPORT/ALPORT_welcome.php).

The mutations in chains α3NC1 and α4NC1 were reported in the publications given as footnotes to the Table.

Protein Change	Protein residue	αNC1
p.Cys1476Phe	C20F	α5NC1
p.Thr1480Arg	T24R	α5NC1
p.Gly1486Ala	G30A	α5NC1
p.Ser1488Phe	S32F	α5NC1
p.Ala1498Asp	A42D	α5NC1
p.Arg1511His	R55H	α5NC1
p.Pro1517Thr	P61T	α5NC1
p.Trp1538Ser	W82S	α5NC1
p.Pro1559Ala	P103A	α5NC1
p.Arg1563Gln	R107Q	α5NC1
p.Cys1564Arg	C108R	α5NC1
p.Cys1564Ser	C108S	α5NC1
p.Cys1567Arg	C111R	α5NC1
p.CYs1567Ser	C111R	α5NC1
p.Glu1568Gln	E112Q	α5NC1
p.Pro1584Leu	P128L	α5NC1
p.Cys1586Arg	C130R	α5NC1
p.Cys1586Phe	C130F	α5NC1
p.Gly1589Val	G133V	α5NC1
p.Trp1590Gly	W134G	α5NC1
p.Gly1596Asp	G140D	α5NC1
p.Tyr1597Cys	Y141C	α5NC1
p.Met1601Ile	M145I	α5NC1
p.Leu1621Ser	L165S	α5NC1
p.Cys1632Gly	C176G	α5NC1
p.Cys1632Trp	C176W	α5NC1
p.Cys1638Tyr	C182Y	α5NC1
p.Leu1649Arg	L193R	α5NC1
p.Ser1659Asn	S203N	α5NC1
p.Arg1677Gln	R221Q	α5NC1
p.Arg1677Pro	R221P	α5NC1
p.Arg1677Leu	R221L	α5NC1
p.Cys1678Arg	C222R	α5NC1
p.Cys1678Trp	C222W	α5NC1
p.Gln1679Pro	Q223P	α5NC1
p.Cys1681Phe	C225F	α5NC1
p.Gly1508Ser[1]	G48S	α4NC1
p.Cys1513Thr[1]	C53T	α4NC1
p.Cys1588Thr[1]	C128T	α4NC1
p.Leu1474Pro[2]	L34P	α3NC1
p.Leu1474Cys[3]	L34C	α3NC1

p.Phe1475Ser[4]	F35S	α3NC1
p.Cys1511Ile[5]	C71I	α3NC1
p.Cys1548Tyr[4]	C108Y	α3NC1
p.Val1550Gly[4]	V110G	α3NC1
p.Trp1578Ser[6]	W138S	α3NC1
p.Qln1495Arg[6]	Q156R	α3NC1

[1] Storey, H., Savige, J., Sivakumar, V., Abbs, S., Flinter, F. A. (2013). J Am Soc Nephrol 24,1945-1954

[2] Lemmink, H. H., Mochizuki, T., van den Heuvel, L. P., Schröder, C. H., Barrientos, A., Monnens, L. A., van Oost, B. A., Brunner, H. G., Reeders, S. T., Smeets, H. J. (1994). Hum Mol Genet 3,1269-1273.

[3]Mochizuki, T., Lemmink, H. H., Mariyama, M., Antignac, C., Gubler, M. C., Pirson, Y., Verellen-Dumoulin, C., Chan, B., Schröder, C. H., Smeets, H. J., Reeders, S. T. (1994). Nat Genet 8,77-81.

[4]Tazón Vega, B., Badenas, C., Ars, E., Lens, X., Milà, M., Darnell, A., Torra, R. (2003). Am J Kidney Dis 42, 952-959.

[5]Heidet, L., Arrondel, C., Forestier, L., Cohen-Solal, L., Mollet, G., Gutierrez, B., Stavrou, C., Gubler, M. C., Antignac, C. (2001). J Am Soc Nephrol 12, 97-106.

[6]Voskarides, K., Damianou, L., Neocleous, V., Zouvani, I., Christodoulidou, S., Hadjiconstantinou, V., Ioannou, K., Athanasiou, Y., Patsias, C., Alexopoulos, E., Pierides, A., Kyriacou, K., Deltas, C. (2007). J Am Soc Nephrol 18, 3004-3016

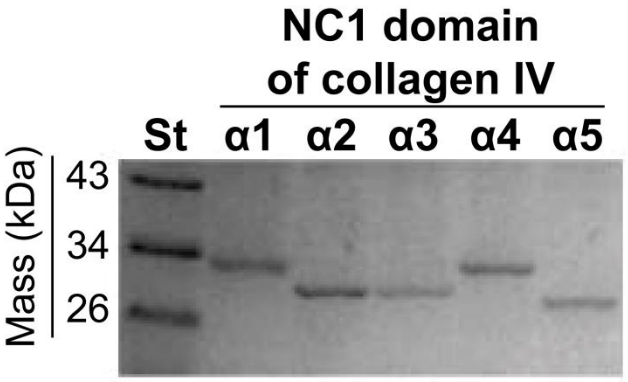

Figure S1 Purified human α1-5NC1 domains produced by recombinant techniques. Coomassie-stained SDS-PAGE (12% polyacrylamide gel). St, protein standards

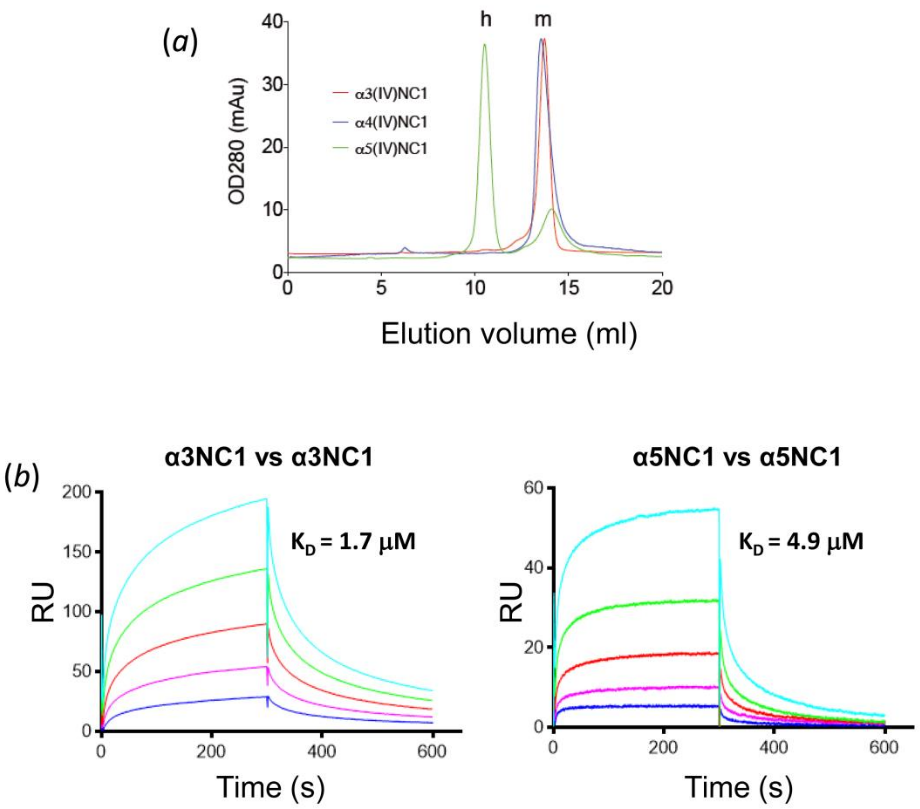

Figure S2 *In vitro* evidence of self-association capacity of α3NC1 and α5NC1. (*a*) Gel filtration chromatography of α3NC1, α4NC1 and α5NC1. "h" and "m" stand for hexamer and monomer, respectively. (*b*) Surface Plasmon Resonace assays. α3NC1 (left) or α5NC1 (right) covalently bound to the sensorchip were used in these assays; the ligands were the α3NC1 (left) or α5NC1 (right) chains in solution at concentrations (profiles from bottom to top) of 0.25, 0.5, 1, 2 and 4 μM. The figure depicts examples of sensorchip traces after subtraction of blanks obtained simultaneously with protein-free solutions. K_D values were estimated from the obtained signal level,(average of two assays at least). For further details, see Materials and Methods.

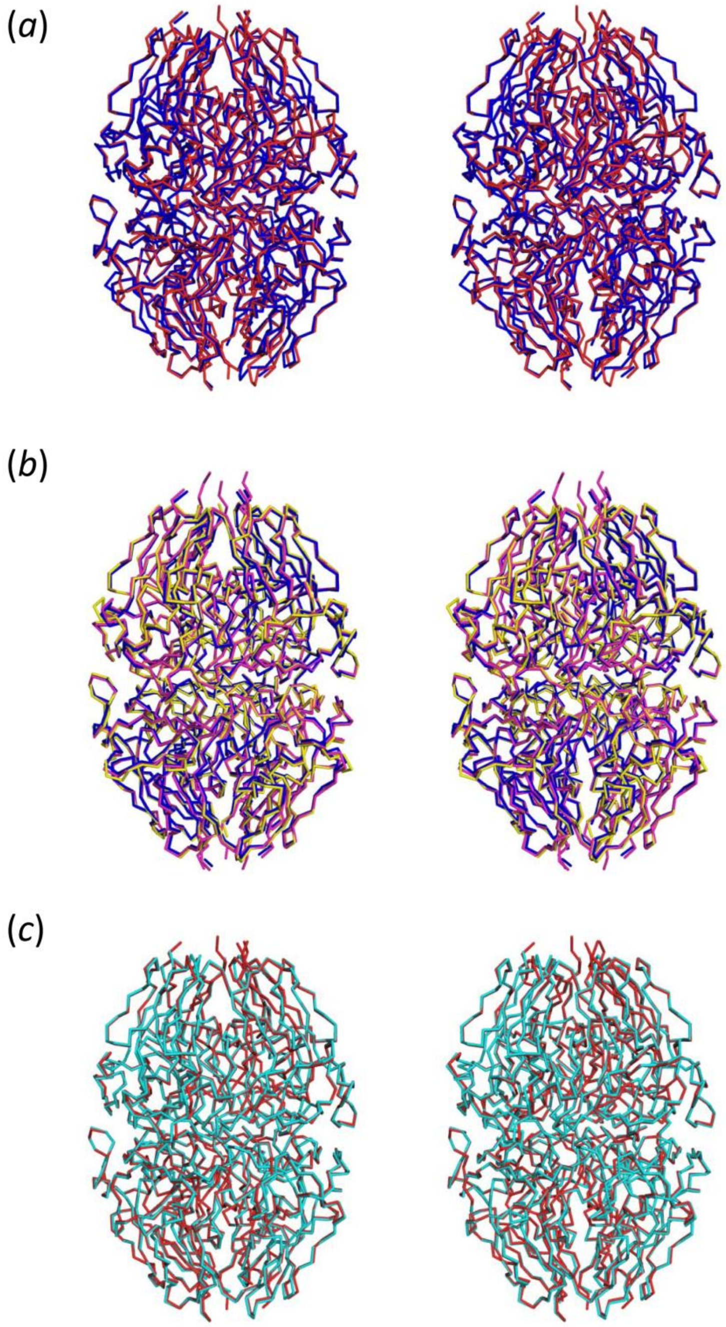

Figure S3 Stereo views of the superimpositions of the backbone of NC1 structures. (*a*) α121NC1 hexamer obtained from human placenta (PDB file 1LI1) (red) superimposed over $\alpha5NC1_{homo}$. (*b*) $\alpha1NC1_{homo}$ (magenta), $\alpha3NC1_{homo}$ (yellow) and $\alpha5NC1_{homo}$ (blue) hexamers. (*c*) α121NC1 hexamers obtained from placenta (red) or produced by recombinant techniques in this study (cyan).

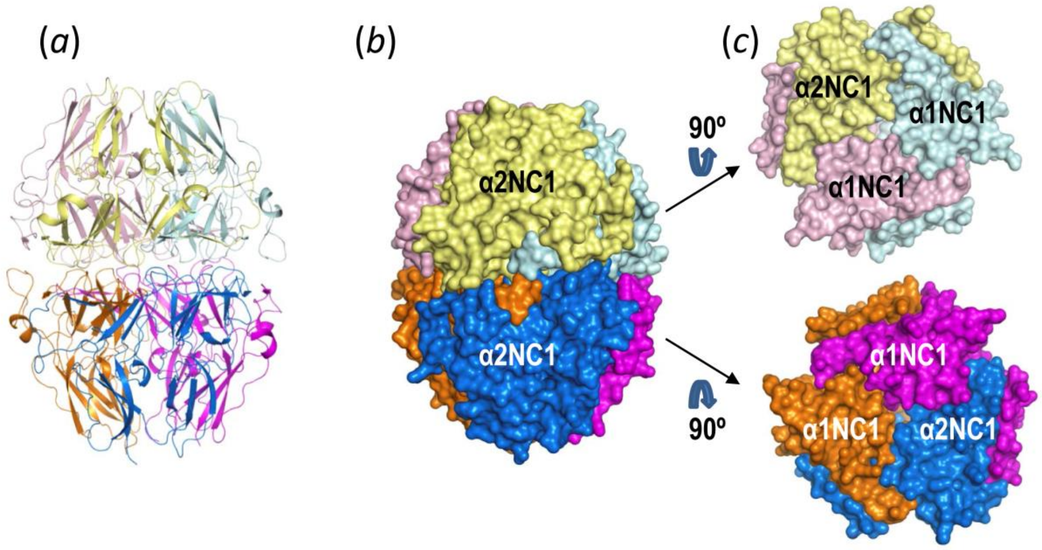

Figure S4 Structure of recombinant α121NC1 found in the asymmetric unit of its crystal: (*a*) cartoon representation or (*b*) surface representation, with each subunit showed with a different colour. In (*c*) the hexamer is deconstructed in its two protomers, viewed from their equatorial surfaces through which both trimers interact.

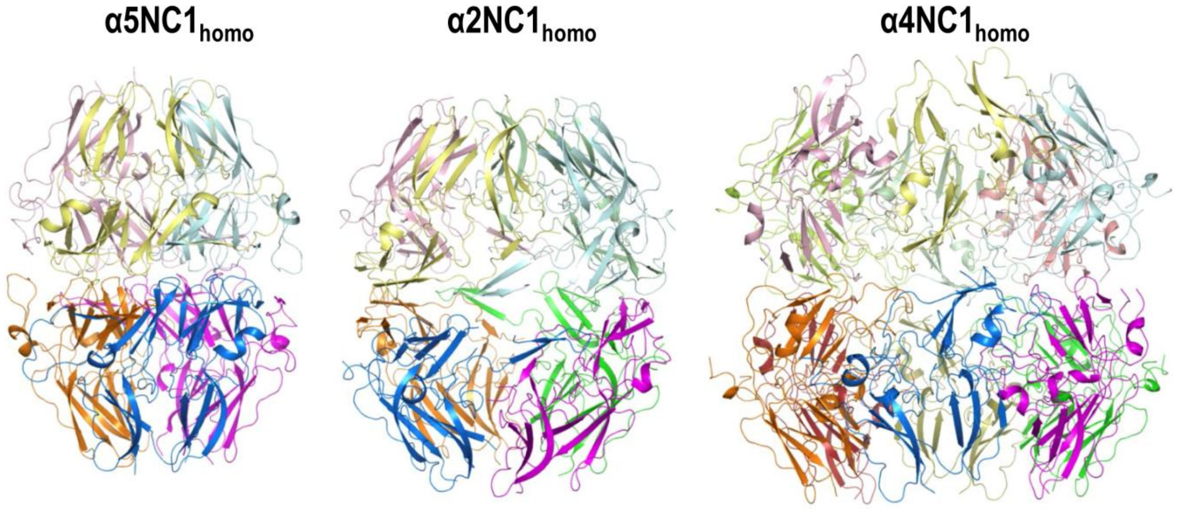

Figure S5

Cartoon representation of the oligomer quaternary structures for the indicated NC1 domains.

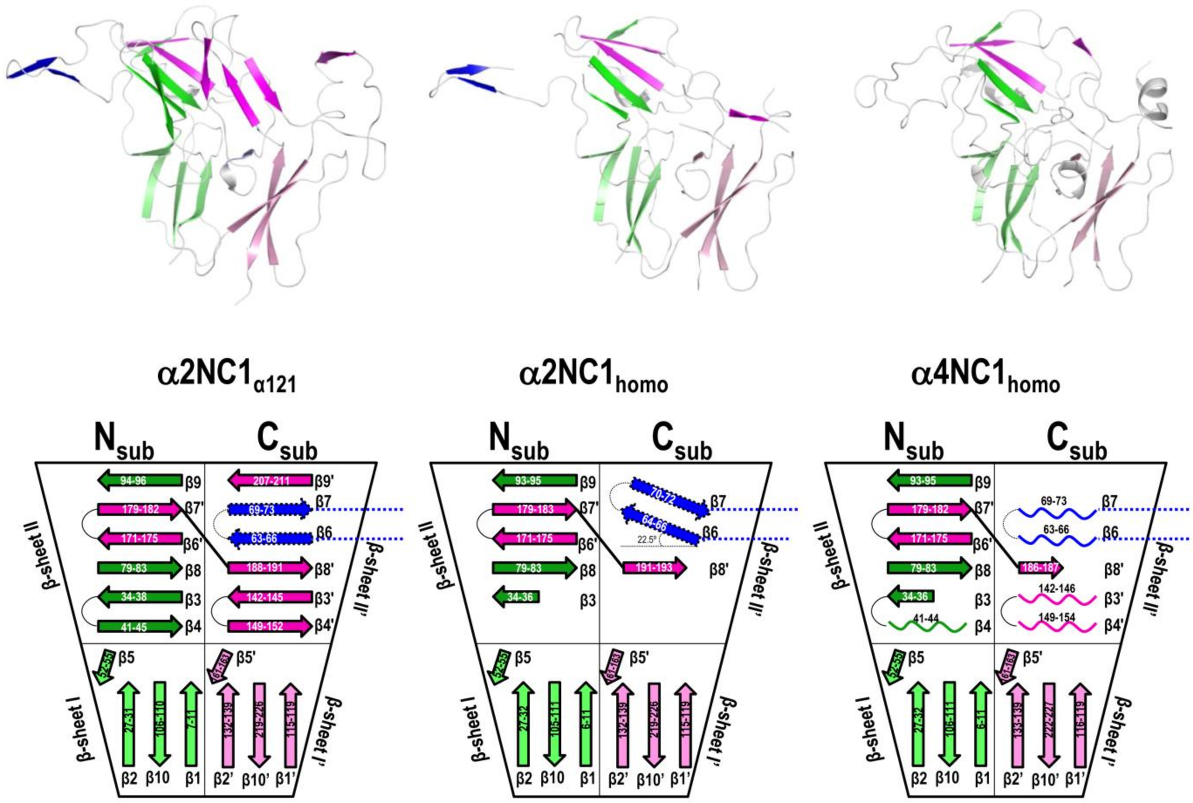

Figure S6 Cartoon representation of the α2NC1 subunit in the α121NC1 hexamer (α2NC1$_{\alpha121}$) and the α2NC1 and α4NC1 domains in the corresponding crystalline homo-oligomers. Below, schemes of the corresponding folds can be seen with the same colour code as in the cartoon representations and labelling of β strands as in Fig 1.

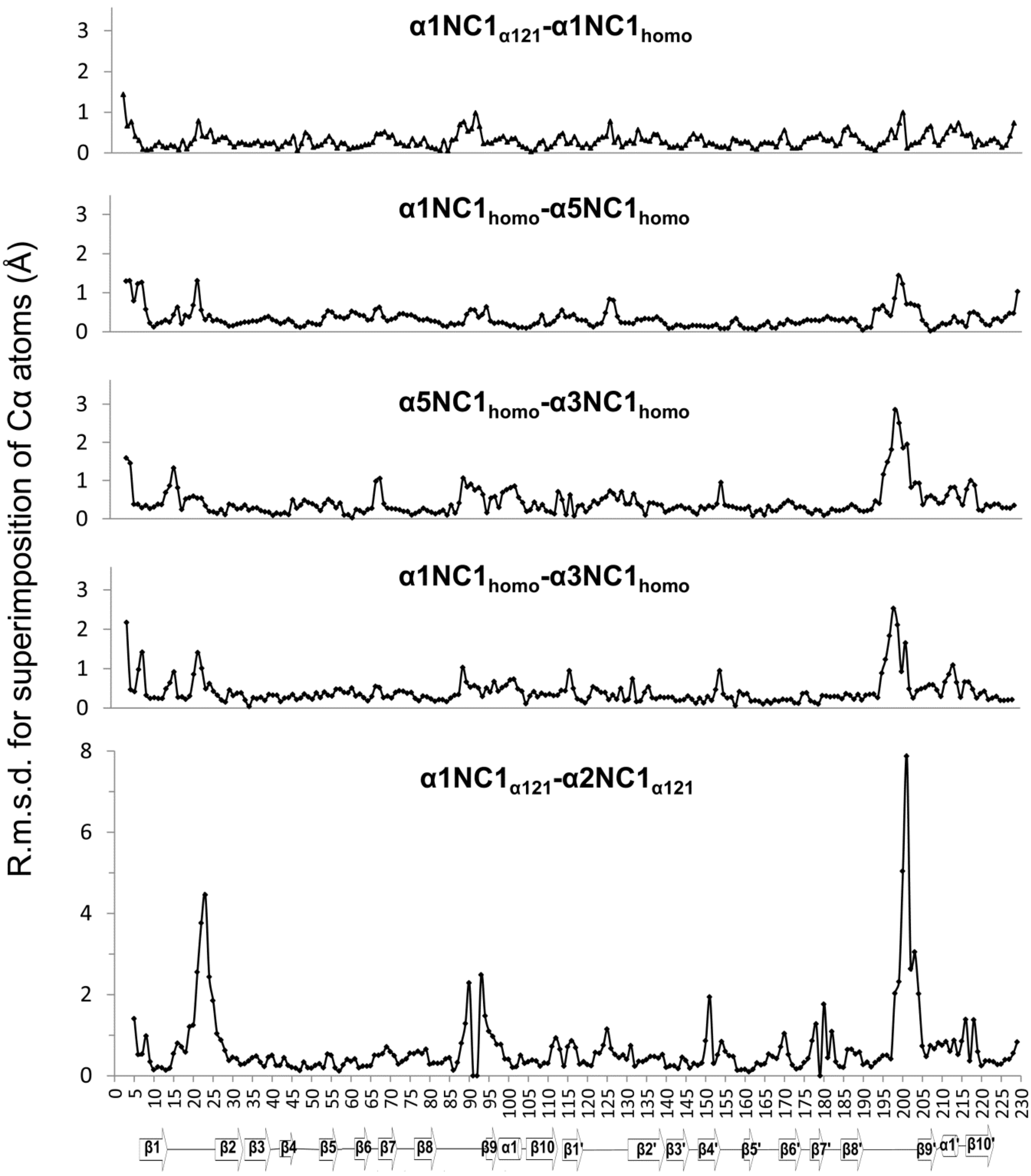

Figure S7 RMSD value for each Cα atom after superposition of the αNC1chains. The residue number and secondary structure of the chains are shown in the X-axis at the bottom.

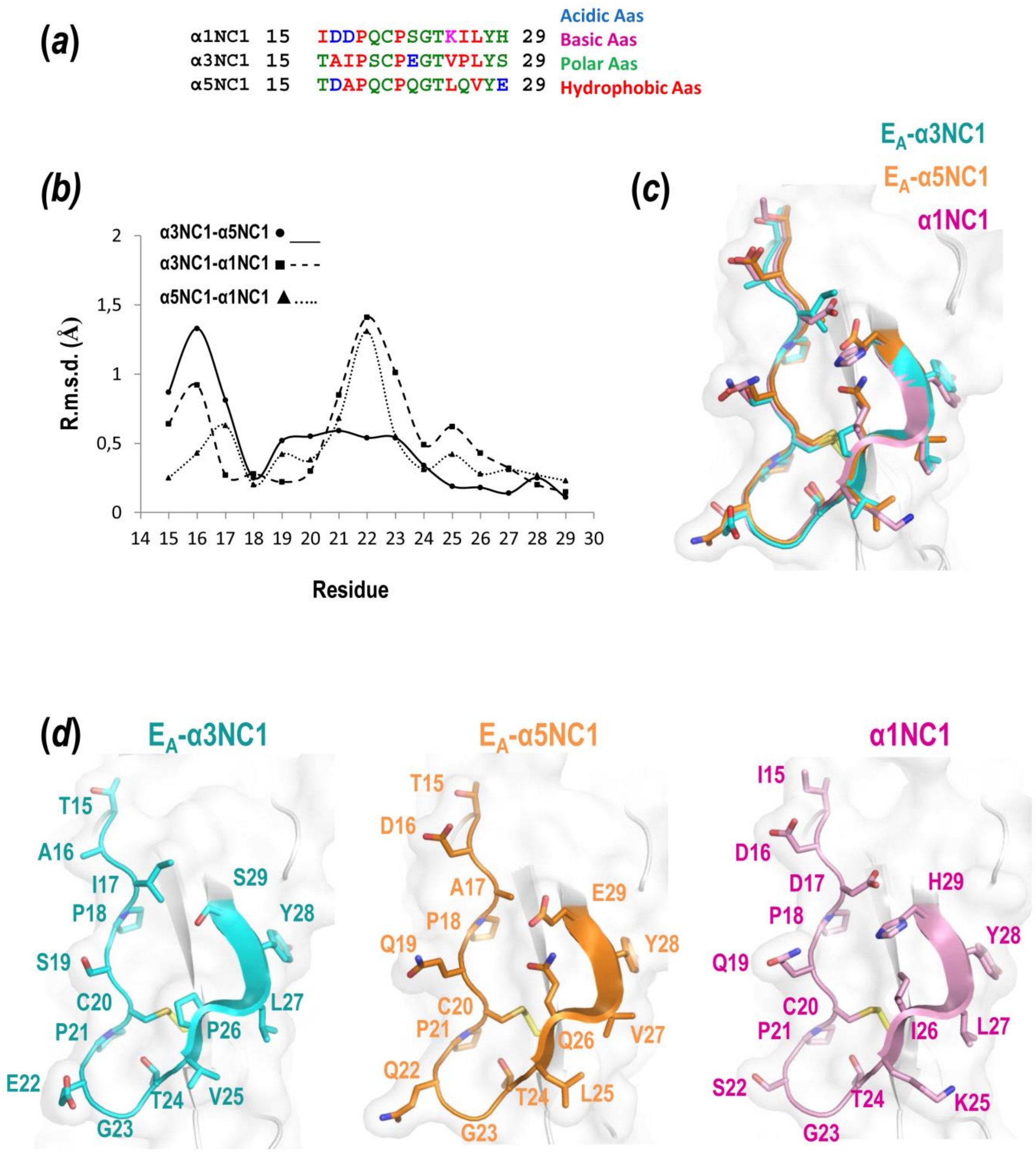

Figure S8 The Goodpasture's E_A epitope. (*a*) Sequence alignment of α3NC1 and α5NC1 (residues 15-29 of the present structures) E_A epitopes with the corresponding residues of α1NC1 (*b*) the RMSD values for the superimpositions of the structures corresponding to the sequences aligned in *a* (all from NC1 domains in homohexamers). (*c*) and (d) Cartoon representation of the structural superposition and their individual structures, respectively.

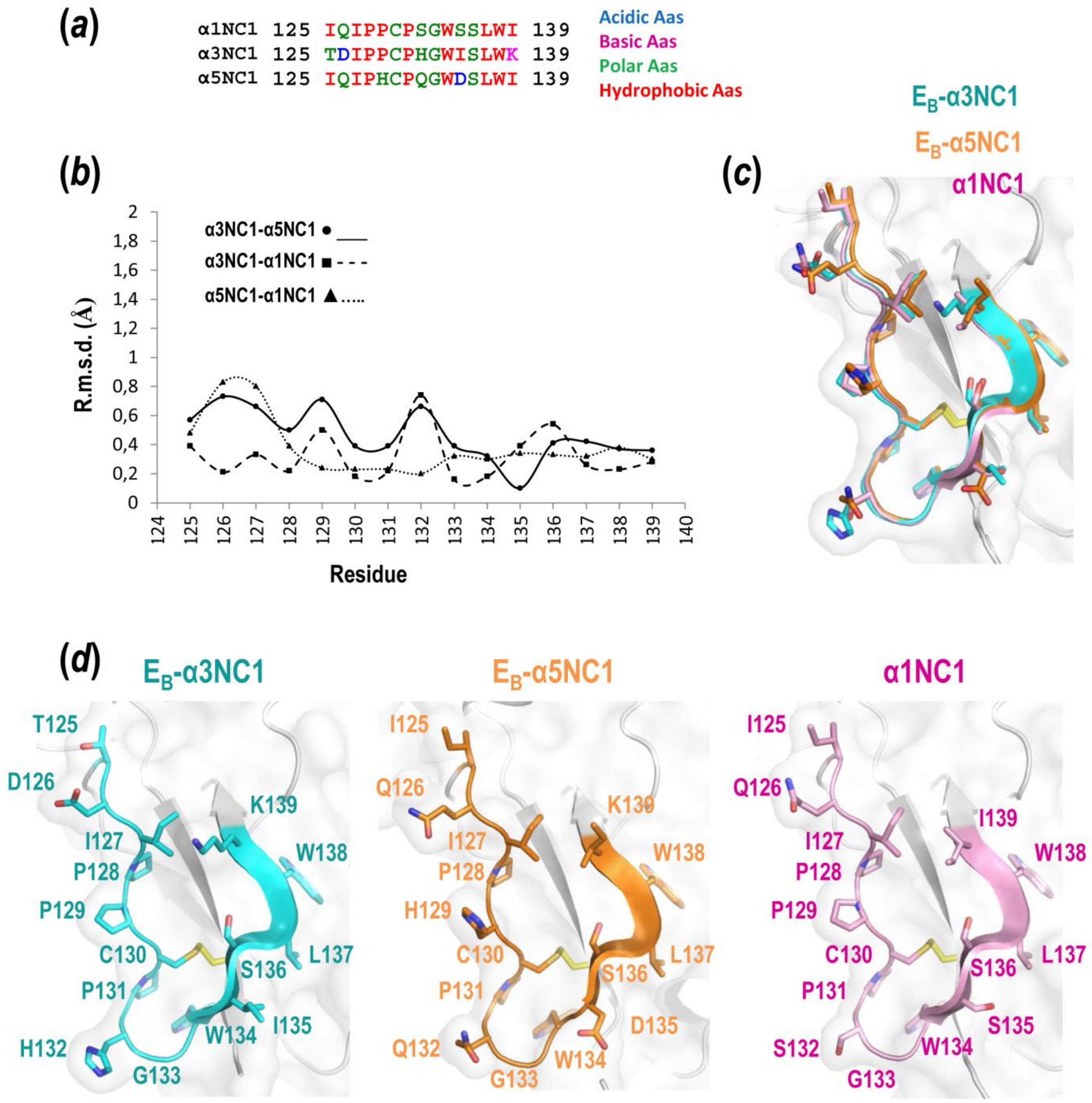

Figure S9 The Goodpasture's E_B epitope. (*a*) Sequence alignment of α3NC1 and α5NC1 (residues 125-139 of the present structures) E_B epitopes, with the corresponding residues of α1NC1 the RMSD values for the superimpositions of the structures corresponding to the sequences aligned in *a* (all from NC1 domains in homohexamers). (*c*) and (d) Cartoon representation of the structural superposition and their individual structures, respectively.

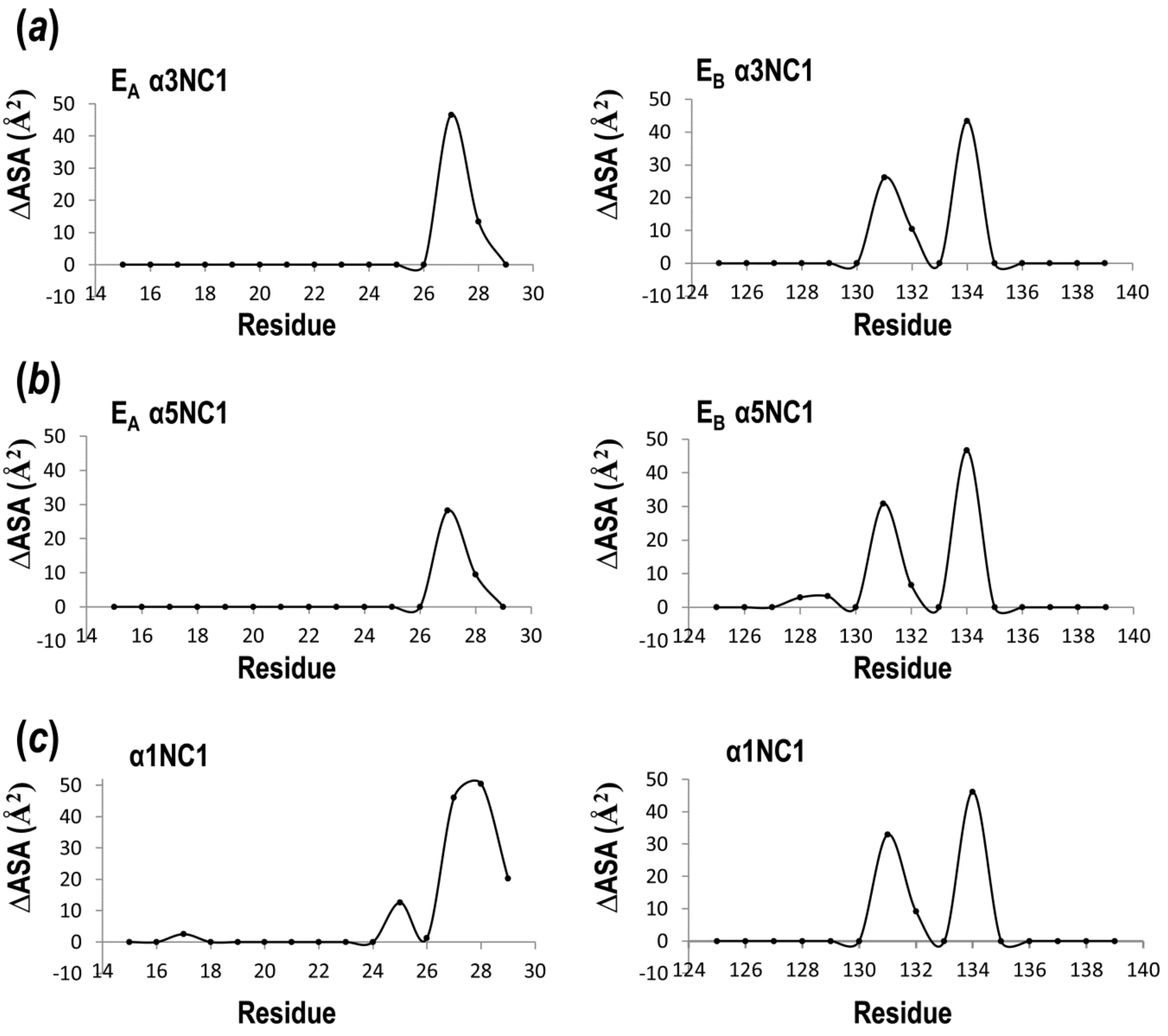

Figure S10 Accessible surface area difference between monomer and homohexamer (ΔASA) for the E$_A$ (left) and E$_B$ (right) epitopes of *(a)* α3NC1 and (*b*) α5NC1 and, for comparison, the corresponding region *(c)* of α1NC1.

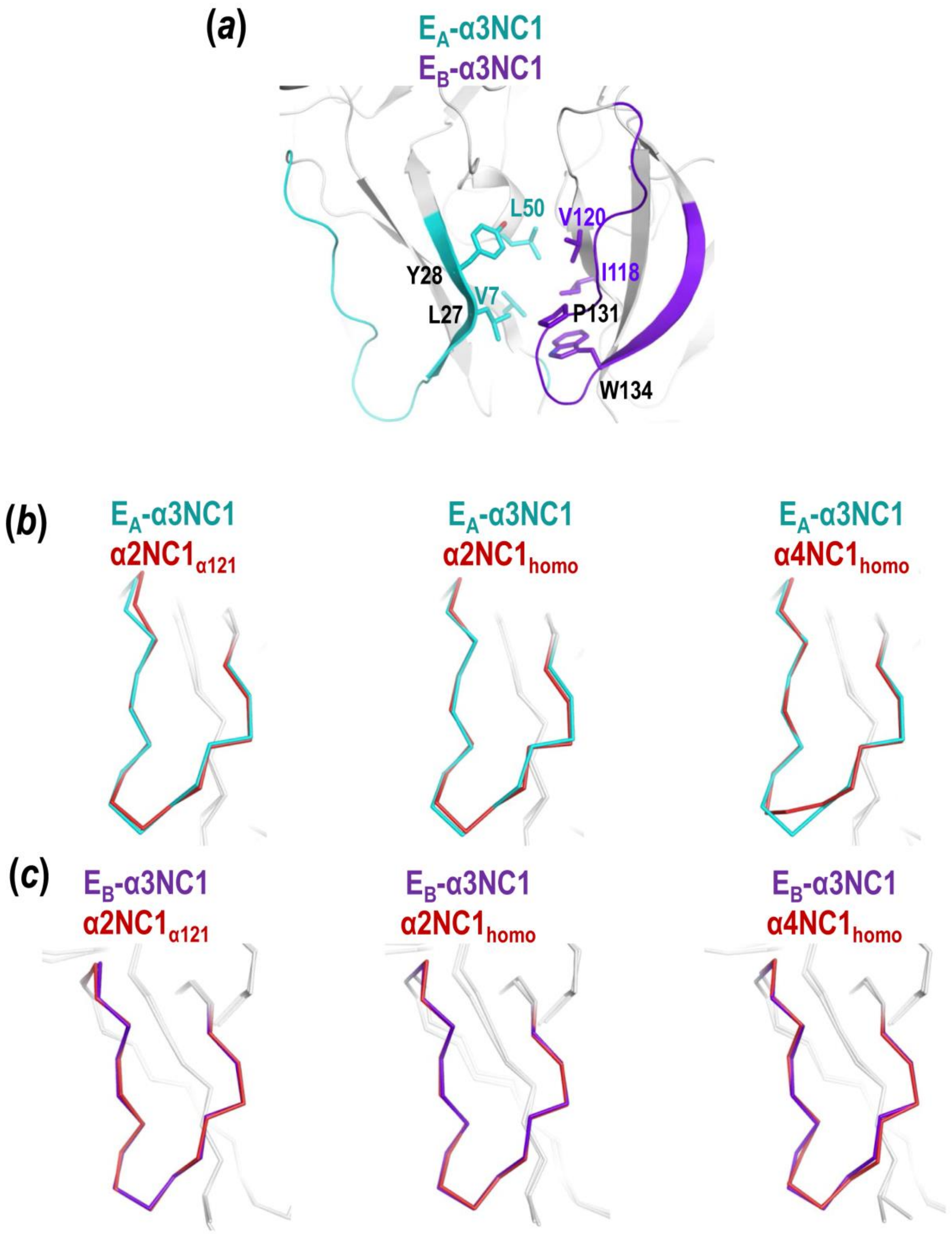

Figure S11 Hydrophobic patch gluing of E_A and E_B epitopes in the α3NC1 domain structure (*a*), and illustration of high structural similarity of the two epitopes of α3NC1 with the corresponding non-autoimmunogenic regions of $α2NC1_{α121}$, $α2NC1_{homo}$ and $α4NC1_{homo}$ (*b*, *c*).
